# Tuning Topologically Nontrivial States in the BHT-Ni Metal–Organic Framework

**DOI:** 10.1021/acs.jpcc.4c06013

**Published:** 2025-01-27

**Authors:** Nafiseh Falsafi, Saeed H. Abedinpour, Fariba Nazari, Francesc Illas

**Affiliations:** † Department of Chemistry, 113403 Institute for Advanced Studies in Basic Sciences, Zanjan 45137-66731, Iran; ‡ Department of Physics, Institute for Advanced Studies in Basic Sciences, Zanjan 45137-66731, Iran; § Center of Climate Change and Global Warming, Institute for Advanced Studies in Basic Sciences, Zanjan 45137-66731, Iran; ∥ Departament de Ciència de Materials i Química Física & Institut de Química Teòrica i Computacional, 16724 Universitat de Barcelona,C/Martí i Franquès 1, 08028 Barcelona, Spain

## Abstract

Using first-principles calculations, we have demonstrated the creation of multiple quantum states in the experimentally accessible metal–organic framework BHT-Ni. Specifically, quantum spin Hall and quantum anomalous Hall states are induced by two- and four-electron doping, respectively. Geometrical symmetry breaking is also investigated in cis- and trans-like structures. For a low electron doping concentration of two electrons per unit cell, the Fermi energy shifts to a nontrivial band gap between the Dirac bands, predicting a quantized spin Hall conductivity. Subsequently at a high electron doping concentration, an anomalous Hall conductivity with a quantized value is observed. In addition, for a centrosymmetric (trans-like) structure, it preserves the quantum spin Hall state and quantized spin Hall conductivity. In contrast, in the noncentrosymmetric (cis-like) structure, the breaking of space inversion symmetry leads to the emergence of the valley Hall effect and the disappearance of spin Hall conductivity.

## Graphical abstract

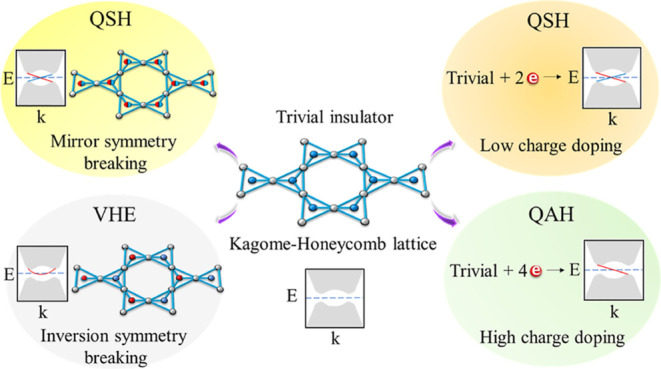

## 1 Introduction

Quantum materials constitute a suitable and information-rich playground for the simultaneous study of topology and symmetry and their mutual effects owing to the profound interplay between them. A diverse and rich spectrum of electronic and topological states and definite symmetry arises from the strong interconnection of lattice-, charge-, spin-, orbital-, and valley degrees of freedom (DOFs).^1–3^ The complex interactions among these DOFs create dynamic interconnections that offer insights into their potential applications.^4^ Among these interactions, spin–orbit coupling (SOC) reveals exciting possibilities for spin devices that operate without magnetic fields.^5,6^ Indeed, in certain systems with strong SOC, topologically protected helical edge or surface states exist.^7^ Along with the bulk electronic energy bands, these states define various quantum or topological phases of matter.^8^

By examining the evolution of band structures under the influence of SOC, numerous topologically interesting phases of matter have been predicted, including the spin Hall effect (SHE), the anomalous Hall effect (AHE), and their quantum counterparts: the quantum spin Hall (QSH) phase and the quantum anomalous Hall (QAH) phase. The SHE, which is the spin version of the Hall effect, produces spin accumulations with opposite orientations on each boundary of the sample,^9^ while the generation of Hall voltage in magnetic materials, resulting from the interaction between magnetization and SOC, refers to AHE. Another exotic state of quantum material is the QSH insulator or two-dimensional topological insulator (2D-TI), where the edges support conducting electronic states (helical edge states) that are protected by time reversal symmetry (TRS). Finally, the QAH insulator is driven by the breaking of TRS due to magnetic polarization.^10^ It is characterized by a finite Chern number, a bulk energy gap, and gapless chiral edge states.^10,11^ This phase can be realized in magnetically doped TIs or intrinsic magnetic topological insulators.^12–14^ Both of the QSH and QAH states are characterized by quantized conductivities like spin Hall conductivity (SHC) and anomalous Hall conductivity (AHC), which appear in the absence of an external magnetic field in contrast to the ordinary Hall effect.^15,16^ These conductivities can be separated into intrinsic parts directly derived from the relativistic band structure^17^ and the extrinsic mechanisms where electrons acquire transverse velocity through skew or side jump scatterings.^9^ The intrinsic AHC and SHC are determined by integrating the ordinary Berry curvature (BC) and spin Berry curvature (SBC) of the occupied bands over the Brillouin zone (BZ), respectively.^18^

In addition to the roles of charge and spin DOFs in electronics and spintronics, the valley DOF introduces the emerging field of valleytronics, which utilizes the valley index of electrons for information storage and manipulation.^13,59^ Among various material platforms, two-dimensional honeycomb lattice structures possess valley DOF alongside charge and spin. However, in the presence of space inversion symmetry (SIS), controlling the valley DOF is challenging.^19^ Breaking SIS opens a finite gap at the Dirac cones, located at the high-symmetry points *K* and *K*′ in the reciprocal space, leading to opposite local BCs at these points.^20^ This symmetry breaking causes electrons in the two valleys to experience opposite effective magnetic fields proportional to the BC. These fields generate Lorentz-like forces, driving electrons from different valleys in opposite directions perpendicular to an applied current. The resulting phenomenon, known as the valley Hall effect (VHE), produces valley-polarized carriers.^19^

While significant efforts have been made to discover new topological materials with exotic phases, the practical application of these properties in devices remains limited. This limitation arises from the slow progress in experimentally synthesizing structures capable of controlling topological phase transitions, which often require extreme tuning conditions or complex multilayered configurations.^21^ In this context, metal–organic frameworks (MOFs) represent a promising class of materials that could significantly broaden the horizons of quantum material design.

In the past decade, significant breakthroughs in manufacturing atomically layered two-dimensional (2D) MOFs with Kagome lattice structures have been reported.^22–24^ Some of these structures have been theoretically predicted and experimentally confirmed^25^ as materials with topological properties, which are known as organic topological materials.^26–31^ The electronic features of a Kagome lattice are Dirac Fermions, which encode information about topology; flat bands (FBs), which are associated with correlated phenomena such as magnetism; and van Hove singularities, which can induce instabilities leading to long-range many-body orders.^32^ Although various lattices demonstrate the presence of FBs, the Kagome lattice distinguishes itself due to its feasibility from a synthetic perspective as it does not require tuning hopping parameters to host a FB.^33^ Note, however, that a FB can be either topologically trivial (FB) or topologically nontrivial; the latter is referred to as a topological flat band (TFB).^34–36^ An isolated TFB can result from strong SOC that induces a nontrivial gap opening,^37^ leading to a nonzero (spin) Chern number.^38^ The emergence of TFB adds further interest to the investigation of topological properties.^39^

Topological phases can be precisely controlled by using an external electric field. This control enables the manipulation of lattice structures, charge distribution, orbital configurations, and spin orientations, providing new opportunities for engineering electronic and topological properties.^40–42^

In the context of organic topological insulators (OTIs), achieving such control is often crucial due to the absence of the Fermi level precisely within the nontrivial band gap. As a result, precise tuning of the Fermi level becomes challenging for identifying and harnessing topologically nontrivial states in these systems.^43^ As an example, the 2D planar MOF, Ni_3_C_12_S_12_, also termed BHT-Ni,^23^ is the first experimentally realized 2D OTI, with its Fermi level located within the trivial band gap. Here, a level of electron doping equivalent to two electrons (per unit cell) of approximately 5 × 10^13^ cm^–2^ ^26,44^ or four electrons (per unit cell), which is equal to ∼2 × 10^14^ cm^–2^, is required to shift the Fermi level into the topologically nontrivial gap.^26^ Doping with electrons or holes has also been proposed for other topological phases.^45,46^ It is essential to highlight that doping primarily adjusts the position of the Fermi level, while the electronic band structure of the OTI remains unaffected.

Additionally, manipulation of the Fermi level has been shown to effectively affect the Hall conductivities. For example, enhancing the SHC, which results from converting charge current into spin current, can be achieved by adjusting the Fermi level.^47^ In addition to SHC, the AHC can also be tuned by altering the chemical potential through electron doping.^28^ Due to the spin-momentum-locked surface states, topological insulators are considered as ideal materials for generating a pure spin current with a large spin Hall angle (SHA), which is the figure of merit for charge-to-spin current interconversion in spin–orbit torque (SOT) devices.^48^ In this regard, finding new materials with a large SHA is decisive.^49^

Based on the previous summary of the existing literature, we present a comprehensive analysis of the electronic, transport, and topological properties of the electron-doped organometallic framework BHT-Ni. We show that at a low electron doping concentration (1.07 × 10^14^ cm^–2^), the trivial insulator BHT-Ni transitions into a QSH insulator, which is characterized by a nonzero Z_2_ topological invariant, while at a higher electron doping level (2.15 × 10^14^ cm^–2^), it transitions into a QAH insulator due to TRS breaking and spontaneous spin polarization, with a finite Chern number of *C* = 1. Altogether, we have analyzed multiple transitions from a trivial insulator^50^ to a QSH insulator, and then to a QAH insulator, all at experimentally accessible doping levels within a single material, confirming the prediction of Zhang et al.^28^ Furthermore, we have identified and analyzed the salient characteristics of topological transport such as SHC and AHC, in pristine and electron-doped BHT-Ni. Finally, the impact of geometrical symmetry breaking on the electronic and topological behavior of centrosymmetric (trans-like) and noncentrosymmetric (cis-like) structures is investigated. These structures are created by substitution of sulfur ligands with iso-valence selenium ligands, which can be experimentally accessed in a manner similar to comparable structures.^51,52^ Through symmetry reduction, the trans-like structure preserves the QSH state and the quantized SHC. In contrast, the breaking of SIS in the cis-like structure leads to the emergence of the VHE and disappearance of the SHC.

## 2 Methods

### 2.1 Computational Details and Material Modeling

The calculation of the electronic structure and topological properties of the BHT-Ni systems examined in this study was performed within the framework of density functional theory^53^ using the Quantum ESPRESSO package.^54^ A plane-wave basis set, augmented with ultrasoft and projector augmented wave pseudopotentials,^55^ was employed in the calculations. The exchange and correlation functional were treated using the generalized gradient approximation of Perdew–Burke–Ernzerhof,^56^ which is widely recognized for its accuracy in describing the electronic structure of materials. The kinetic energy cutoff for the plane-wave basis was chosen to be 80 Ry. A (3 × 3 × 1) ***k***-point mesh was used for BZ sampling. The SOC effect was taken into account for the calculation of the electronic structures and topological properties. We also calculated the band structures using a PBE + U functional and considering various U values, including U = 0, 3, and 5 eV, with the corresponding results presented in Figure S1. As observed, the band gap and the target Kagome bands remain largely unaffected by the inclusion of the U parameter, demonstrating that the choice of U has a minimal impact on the overall properties of the target bands.

Models for pristine, low, and high electron doping concentrations and cis- and trans-like configurations were used, which are denoted as (BHT-Ni)_p_, (BHT-Ni)_l_, and (BHT-Ni)_h_ and *cis*-(BHT-Ni)_p_, *cis*-(BHT-Ni)_l_, *trans*-(BHT-Ni)_p_, and *trans*-(BHT-Ni)_l_, respectively. To avoid distortions in hexagonal symmetry and ensure consistency for comparison when designing cis- and trans-like structures, we rely on the high symmetry subgroups of D_6h_. In the structural optimization, the atomic positions were relaxed until the forces on all atoms were smaller than 0.002 Ry/Bohr. In our first-principles calculations, the doping effect is simulated by adding electrons to the lattice and meanwhile adding a homogeneous background charge of opposite sign to maintain the system charge neutrality.

To investigate the stability of the (BHT-Ni)_p_ monolayer structures, we calculated the cohesive energy (E_C_) as

$$E_{C}=\frac{\left[E_{{\text{Ni}}_{3}C_{12}X_{a}Y_{b}}-\left(N_{\text{Ni}}E_{\text{Ni}}+N_{C}E_{C}+N_{X}E_{X}+N_{Y}E_{Y}\right)\right]}{N}$$ 1

where $E_{{\text{Ni}}_{3}C_{12}X_{a}Y_{b}}$ is the total energy of the monolayer of interest, and *E*_Ni_, *E*_C_, *E*_*X*_, and *E*_Y_ are the total energies of the free Ni, C, X, and Y atoms, respectively. In the (BHT-Ni)_p_ structure, X = Y= S, while in the *cis*- and *trans*-(BHT-Ni)_p_ structures, X = S and Y = Se. N_Ni_, N_C_, N_X_ = a, and N_Y_ = b are the number of Ni, C, X, and Y atoms, respectively. N stands for the total number of atoms in the monolayer structures. The cohesive Energy (*E_C_*) of all models including (BHT-Ni)_p_, (BHT-Ni)_l_, (BHT-Ni)_h_, *cis*-(BHT-Ni)_p_, *trans*-(BHT-Ni)_p_ are reported in Table 1.

**Table 1:** Cohesive Energy (*E*_C_) Per Atom (eV) of (BHT-Ni)_p_, *cis*-(BHT-Ni)_p_, and *trans*-(BHT-Ni)_p_, (BHT-Ni)_l_ and (BHT-Ni)_h_ Compared to Graphene

structure	graphene	(BHT-Ni)_p_	*cis*-(BHT-Ni)_p_
EC/atom	–5.65	–6.39	–5.94
structure	*trans*-(BHT-Ni)_p_	(BHT-Ni)_l_	(BHT-Ni)_h_
*E*_C_/atom	–5.95	–6.53	–5.63

The Bloch states obtained from the periodic calculations are subsequently expressed in the Wannier basis by projecting onto p_*z*_ orbitals of C, S, and Se as well as the d_*xz*_ and d_*yz*_ orbitals of Ni, which collectively form the Kagome bands near the Fermi level. The Wannierization based on a set number of target bands is a widely employed method for studying topological properties.^57–60^ The choice of orbitals for these projections, informed by the projected density of states (PDOS) and the orbital-resolved band structures, ensures a precise and comprehensive representation of the electronic structure across all compounds, (BHT-Ni)_p_, (BHT-Ni)_l_, (BHT-Ni)_h_, *cis*-(BHT-Ni)_p_, *cis*-(BHT-Ni)_l_, *trans*-(BHT-Ni)_p_, and *trans*-(BHT-Ni)_l_. The initial projected Wannier functions are then optimized to obtain maximally localized Wannier functions (MLWFs) using the WANNIER90 code.^61^ The MLWFs are then used to derive the tight-binding Hamiltonian in these localized bases.^62^ Utilizing the postprocessing module (BERRY) of the WANNIER90 code developed by Qiao et al.,^18^ the SHC and SHA are calculated. The SHC that appears in the Kubo formula approach (see next part) is achieved by integrating the Berry-like curvature over the BZ.^8,18^ For the Wannier interpolation, we chose a 500 × 500 × 500 ***k***-mesh grid for SHC calculations. An adaptive refinement ***k***-mesh of 4 × 4 × 4 was used in the integral calculations. The method of adaptive ***k***-mesh refinement can be effective for an efficient convergence of SHC calculation.^18^ The semi-infinite edge states were calculated using a real-space Hamiltonian in the basis of MLWFs and the iterative Green’s function approach implemented in WannierTools.^63^

Using the provided tools and the principles detailed in the next section, we examined key topological properties such as SHC, AHC, SBC, BC, and SHA, offering insights into the relationship between the electronic structure and topological effects in quantum materials. These investigations provide the groundwork for understanding the charge or spin conductivities that connect applied electric fields to induced charge or spin currents, which can be compactly expressed as *J*_α_^γ^ = σ_αβ_^γ^ *E*_β_, where the lower indices α and β refer to the spatial directions of the induced current and applied field, respectively. The upper index, γ, refers to the charge DOF (γ = *c*) for the charge conductivity or to the spin-polarization direction (γ = *x*, *y*, *z*) for the spin conductivity. Different components of the conductivity tensor σ_αβ_^γ^ could be calculated by using the Kubo formula. In the modern language, the Kubo formula for the transverse conductivities in the direct current (dc) limit is conveniently expressed in terms of the BC and SBC,^64^ as

2 $$\sigma_{\alpha \beta }^{\gamma }=-\frac{e^{2}}{\hbar }\int_{BZ}\frac{dk}{{\left(2\pi \right)}^{d}}\Omega_{\alpha \beta }^{\gamma }\left(k\right)$$

here, *d* is the dimension of the system and the total ***k***-resolved (ordinary and spin) BCs Ω_αβ_^γ^(***k***) are

3 $$\Omega_{\alpha \beta }^{\gamma }\left(k\right)=\sum_{n}f\left(E\right)\Omega_{n,\alpha \beta }^{\gamma }\left(k\right)$$

where the sum is over bands *n*, *f* (*E*) = 1/[*e* ^(*E*–μ)^/(*k*_B_T) + 1] is the Fermi–Dirac distribution function with μ, *k*_B_, and *T*, the chemical potential, Boltzmann constant, and absolute temperature, respectively. The Fermi–Dirac factor restricts the sum to the occupied bands. The band-projected BC-like term is defined as^18^

4 $$\Omega_{n,\alpha \beta }^{\gamma }\left(k\right)={-2\hbar }^{2}Im\sum_{m\neq n}\frac{\left\langle nk\left|{Ĵ}_{\alpha }^{\gamma }\right|mk\right\rangle \left\langle mk\left|{\mathrm{\nu ̂}}_{\beta }\right|nk\right\rangle }{{\left(\epsilon_{nk}-\epsilon_{mk}\right)}^{2}}$$

where ϵ_*n* ***k***_ and ϵ_*m* ***k***_ are the eigenvalues corresponding to the Bloch eigenstates |*n* ***k*** ⟩ and |*m* ***k*** ⟩. The spin-velocity operator is defined as ${Ĵ}_{\alpha }^{\gamma }=\left\{\sigma_{\gamma },{\mathrm{\nu ̂}}_{\alpha }\right\}/2$ , where ${\mathrm{\nu ̂}}_{i}=\hbar^{-1}\partial Ĥ/\partial k_{i}$ with *i* = α and β representing the velocity operator (Ĥ is the Hamiltonian), and σ_γ_ with γ = *x*, *y*, and *z* are the Pauli matrices. Taking σ_*c*_, a (2 × 2) identity matrix, we find ${Ĵ}_{\alpha }^{c}={\mathrm{\nu ̂}}_{\alpha }$ and can define ordinary BCs and SBCs in the same manner. Although the third-rank tensor σ_αβ_^γ^ can, in principle, have several components, symmetry constraints fix the number of its independent elements^65,66^ Recently, symmetry is utilized to determine the nonzero components of the SHC tensor and simplify the calculations in Weyl semimetals^47^ and topological insulators.^67^ According to the symmetry analysis, the allowed independent SHC components for all 230 space groups are tabulated in Table S1 .^65^ On the other hand, in two spatial dimensions, which is the focus of our study here, charge and spin Hall conductivities each can have at most a single independent nonvanishing element, i.e., σ_*xy*_^*c*^ = −σ_*yx*_^*c*^ and σ_*xy*_^*z*^ = −σ_*yx*_^*z*^. Note that the unit of BC Ω_αβ_^γ^(***k***) is length^2^, and therefore in the way we have defined σ_αβ_^γ^ through eq 2, the units of both AHC and SHC reads *e* ^2^/ℏlength^(2–*d*)^ (*d* = 2, 3 is dimension of the system). While this is the standard unit for charge conductivities, to convert the SHC into its conventional units *e* length^(*d*–2)^, it should be scaled by – ℏ/(2*e*) (note that *e* ^2^/ℏ ≃ 2.434 × 10^–4^ S). The ab initio calculation of topological conductivities based on the Wannier method was first introduced by Wang et al.^68^ for the AHC. Subsequently, this method was expanded to examine the SHC as well. To evaluate the SHA here, we have adapted a two-dimensional version of the θ_SH_ ^18^

5 $$\theta_{\text{SH}}=\frac{2e}{\hbar }\left(\frac{\sigma_{xy}^{z}}{\sigma_{yy}}\right)$$

the longitudinal electrical conductivity (EC), σ_*yy*_, should be also calculated. To this end, we used the semiclassical Boltzmann transport equation^69^

6 $$\sigma_{\alpha \beta }\left(\mu ,T\right)=e^{2}\int_{-\infty }^{\infty }dE\left(-\frac{\partial f\left(E\right)}{\partial E}\right)\sum_{\alpha \beta }\left(E\right)$$

here

7 $$\sum_{\alpha \beta }\left(E\right)=\int \frac{dk}{{\left(2\pi \right)}^{d}}\nu_{\alpha }\left(n,k\right)\nu_{\beta }\left(n,k\right)\tau_{n,k}\delta \left(E-E_{n,k}\right)$$

is the transport distribution function and ⟨*n* ***k*** |ν_i_|*n* ***k*** ⟩, where i = α,β is the band velocity. Moreover, τ_*n*, ***k***_ is the relaxation time, which generally depends on the band index and wave vector. However, we resort to the constant relaxation time approximation, i.e., τ_*n*, ***k***_ = τ, throughout this work.

### 2.2 Crystal Structure and Main Symmetry Features

A benzene hexathiol (BHT) molecule consists of a benzene core and three chelating dithiolene ligands. The symmetric structure of the benzene core, combined with the usefulness of covalent metal-dithiolene bonding, makes it an exceptional molecular building block for the construction of porous polymer MOFs, such as benzene hexathiol-Ni (BHT-Ni), which was achieved through a coordination reaction between BHT and nickel­(II) acetate (Ni-(OAc)_2_).^23^ In BHT-Ni, which we refer to as (BHT-Ni)_p_, each BHT unit bonds with three Ni atoms to form a structurally perfect Kagome lattice pattern, illustrated by the blue dashed lines in the Figure 1a. (BHT-Ni)_p_ that is also known as Ni-bis-dithiolene Ni_3_(C_6_S_6_)_2_, and exhibits a planar configuration and 6-fold symmetry as a result of structural relaxation. In addition, first-principles simulations demonstrated its thermal stability^70^ and dynamical stability.^71^ The metal sites in these M_3_L_2_-type MOFs have a local structure of four neighboring atoms in a planar configuration, regardless of the type of ligand molecules. For example, the Ni atom in (BHT-Ni)_p_ (Figure 1a) is surrounded by four sulfur atoms.^72^ The (BHT-Ni)_p_ structure is classified as a hexagonal crystal with SIS. The symmetry of the crystal corresponds to the *P*6/*mmm* (no. 191) space group, whose point group is D_6h_. Its crystal structure is generated by a 3-fold rotational symmetry (C_3*z*_), the vertical mirror symmetry—because of the 3-fold rotational symmetry, there are three equivalent vertical mirror symmetries *M*_*vi*_ (*i* = 1, 2, and 3), as shown in Figure 1b— horizontal mirror symmetry (*M*_h_), and the SIS.

**Figure 1:**
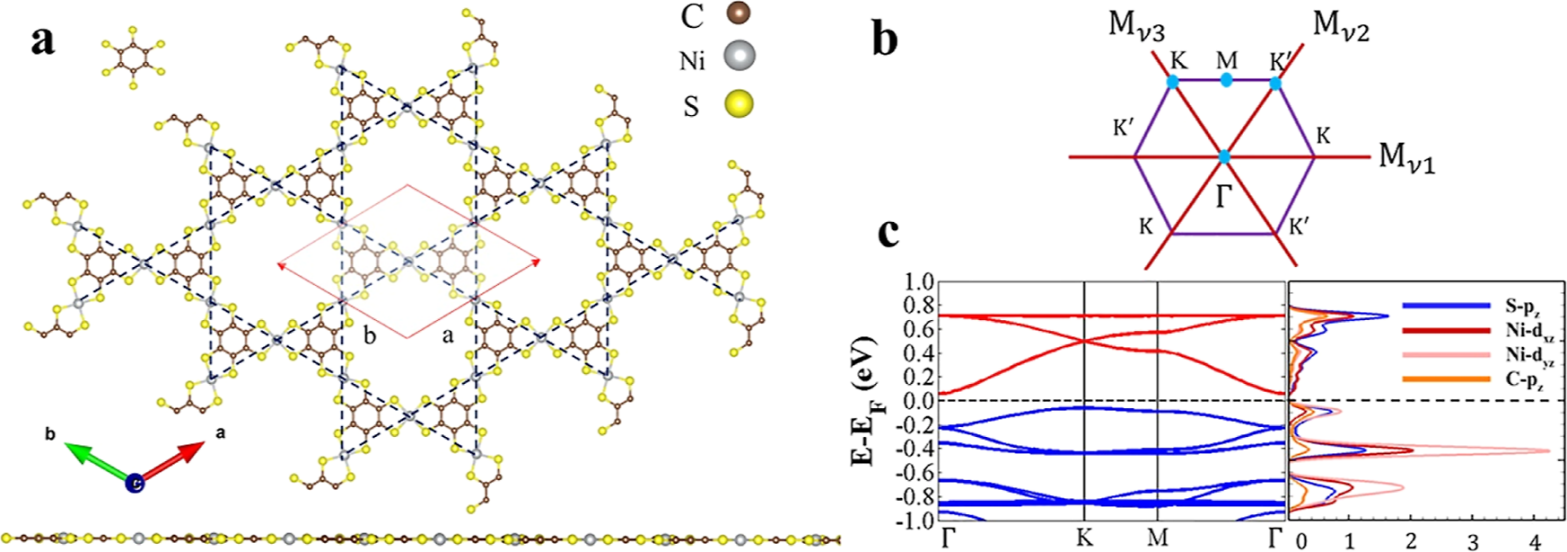
(a) Atomic structure of (BHT-Ni)_p_ represents the Kagome lattice outlined by blue dashed lines. The red solid lines illustrate the unit cell structure with *a* and *b* lattice vectors. The top-left figure shows the motif (BHT) that forms the unit cell. The bottom panel shows the planar optimized structure of the 2D MOF (BHT-Ni)_p_, with C atoms in brown, Ni in gray, and S in yellow. (b) The first BZ and three vertical mirror planes. (c) Band structure without SOC, showing the red Kagome bands and the PDOS for (BHT-Ni)_p_. The energy at the Fermi level is set to zero.

The unit cell of (BHT-Ni)_p_, depicted with red lines in Figure 1a, includes 3 Ni atoms, 12 C atoms, and 12 S atoms. This 2D d^8^ planar transition metal compound, (BHT-Ni)_p_, is known to exhibit efficient π–d conjugation, leading to delocalized electrons throughout the structure.^50^ The optimized lattice constants of the (BHT-Ni)_p_ monolayer are *a* = *b* = 14.64 Å, which agree well with recent experimental measurements^22^ and previous first-principles DFT results.^26,70^ The calculated band structure excluding SOC, and the corresponding PDOS, is shown in Figure 1c. The agreement between the results from present DFT calculations for the crystal structure and the previously reported data^26,44,57,70,73^ confirms the suitability of the chosen computational methods and parameters to proceed the investigation.

## 3 Results and Discussion

### 3.1 Electronic and Topological Properties of Pristine and Low Electron Doping Regime in BHT-Ni

Before proceeding with the results, it is important to highlight that the studied structure (BHT-Ni)_p_ was confirmed to be thermodynamically stable. The cohesive energy values (eq 1), which underscore this stability, are reported in Table 1 and have been systematically compared with available data to ensure the accuracy and reliability of our results. Additionally, the stability of the studied structures has been compared to that of graphene, serving as a benchmark to further validate our findings.

As all of the calculations for the structures described in the computational section rely on the properties of the pristine structure, we will first provide a detailed account of the calculations and results obtained for (BHT-Ni)_p_. Subsequently, we will present the results and analyses for the corresponding charged structures. Building on the aforementioned details, (BHT-Ni)_p_ is a semiconductor with a narrow indirect energy gap of 0.122 eV. Figure 1c illustrates that the Kagome bands (cf. red bands in Figure 1c left) comprise an FB positioned above the two Dirac bands. These bands are situated above the Fermi level (*E*_F_) within the energy range of *E*_F_ < *E* < *E*_F_ + 0.8 eV.^44,57,73^ These energy bands resemble those of graphene, featuring a Dirac cone at the *K* point, degenerate with an FB at the Γ point. However, it is important to note that these degeneracies are removed by SOC, resulting in the emergence of the TFB (see Figure S2a). In fact, incorporating SOC into (BHT-Ni)_p_, reveals a global energy gap of Δ_3_ = 5.4 meV and a local energy gap of Δ_2_ = 17 meV at the Γ point, as well as a Dirac energy gap of Δ_1_ = 14 meV at the *K* point. These values align well with recent first-principles results, which report a global gap in the range of Δ_3_ = 4–5.8 meV,^26,57^ Δ_2_ = 17 meV^44^ for the local gap at the Γ point, and Δ_1_ = 13.6–14 meV for the Dirac gap.^26,44,58^ These gaps are mainly attributed to intrinsic SOC within the d-orbitals of Ni atoms. Notably, the Rashba SOC effect can be excluded due to the presence of inherent SIS. These SOC gaps are much larger than the gap observed in graphene.^74^ The enhanced SOC interactions here are attributed to the hybridization of the p_*z*_ orbitals of light atoms with the d-orbitals of Ni atoms. The SOC gaps of OTIs typically fall within this range (2.3–255 meV).^45,75^ The paired Ni-3d electrons preserve the TRS of the original (BHT-Ni)_p_ structure. Consequently, a nonmagnetic ground state is predicted,^72^ with the SOC facilitating the emergence of the QSH phase.

As previously stated, having the Fermi level (*E*_F_) precisely situated within the nontrivial gap of the material is beneficial for experimental measurements of electronic transport properties. Therefore, we tried to raise *E*_F_ to the Kagome band region through electron doping. Based on first-principles calculations at a doping concentration of 1.07 × 10^14^ cm^–2^, corresponding to two additional electrons per unit cell, the *E*_F_ shifts exactly to the Dirac point, without changing the Kagome bands, as evident by comparison of Figure 2a,b. The projected band structure of (BHT-Ni)_l_, in Figure 2c illustrates that electron doping does not change the nature of the Kagome bands [cf. Figure S3 shows the projected band structure of (BHT-Ni)_p_]. With electron doping, the lattice constants increased slightly, by approximately 0.9%, but both symmetries, TRS (*E* (***k***, ↑) = *E* (−***k***, ↓)) and SIS (*E* (***k***, ↑) = *E* (−***k***, ↑)), are effectively retained. This ensures that the energy bands retain their spin degeneracy, known as Kramers’ degeneracy.^76^ Notably, this doping regime is achievable under experimental conditions.^77^

**Figure 2:**
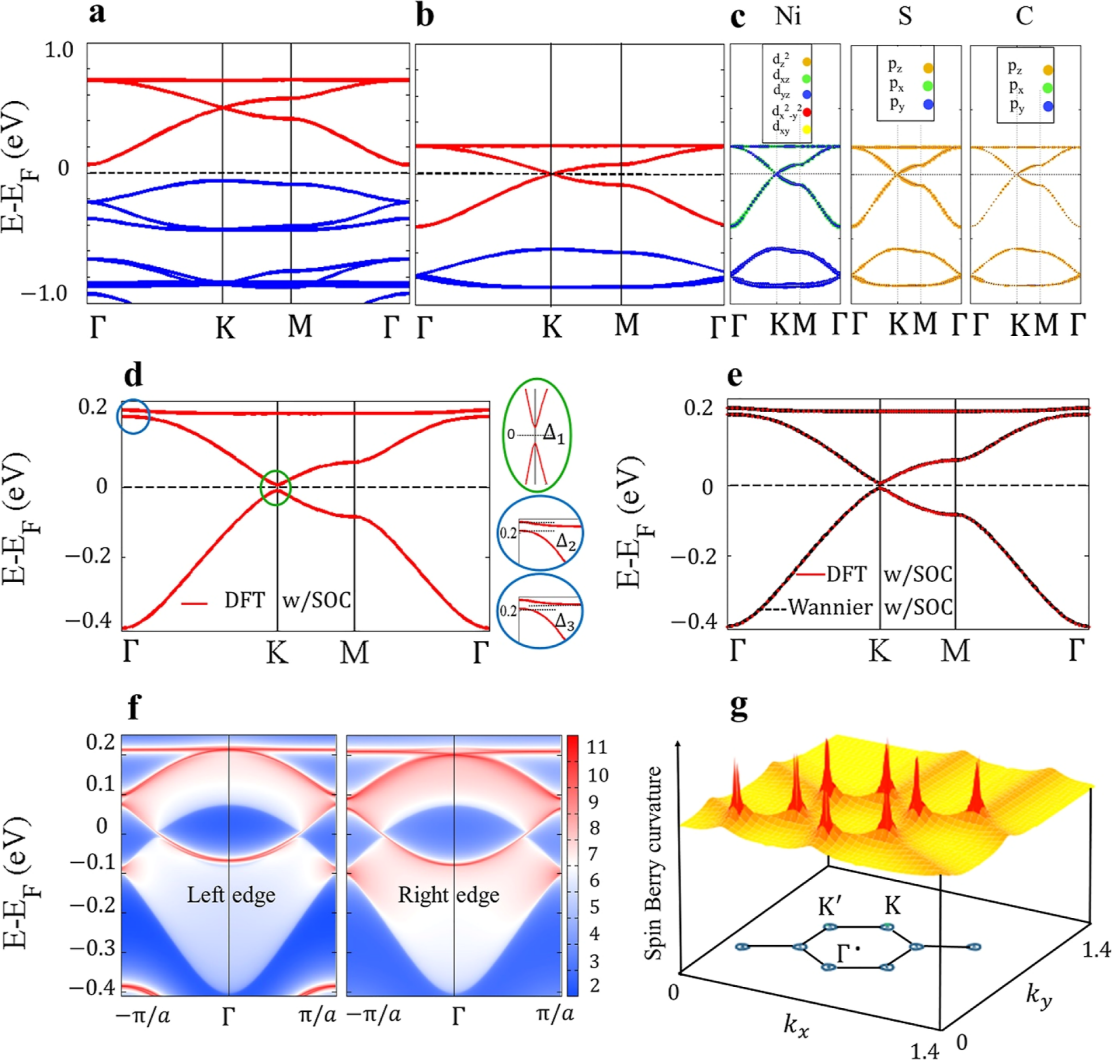
(a) Band structure of (BHT-Ni)_p_ compared to (b) that of (BHT-Ni)_l_ at an electron doping concentration of 1.07 × 10^14^ cm^–2^, calculated using DFT without SOC, (c) orbital-resolved (projected) band structure of the Kagome bands of (BHT-Ni)_l_, projected onto the Ni, S, and C atoms, respectively, and (d) band structure of (BHT-Ni)_l_ with SOC and its SOC gaps (Δ_1_,Δ_2_, and Δ_3_). (e) DFT and MLWF-fitted band structures with SOC. (f) Semi-infinite helical edge states within the SOC gaps. (g) ***k***-resolved SBC on the ***k***_*x*_–***k***_*y*_ plane in the BZ at *E* = *E*_F_ by considering SOC.

The DFT calculations, including SOC, reveal that the degeneracies of the two Dirac bands at the *K* point as well as those between the upper Dirac band and the TFB at the Γ point are removed. This results in energy gaps of Δ_3_ = 8.5 meV as the global gap, Δ_2_ = 18.6 meV as the local gap at the Γ point, and Δ_1_ = 15.4 meV as the Dirac gap at the *K* point (see Figures 2d and S2b).

To investigate the topological properties, we calculated the edge states, Z_2_ invariant, and the BC of the (BHT-Ni)_p_ and (BHT-Ni)_l_ structures. Owing to the presence of a trivial gap in the case of (BHT-Ni)_p_, no edge states at the Fermi level were observed, and the topological Z_2_ invariant was found to be zero. However, (BHT-Ni)_l_ characterized by a nontrivial gap at the Fermi level is anticipated to support a nontrivial topological phase. Figure 2e depicts the fitted band structure of (BHT-Ni)_l_ with Wannier interpolation, in the energy window of (*E*_F_ – 0.5, *E*_F_ + 0.4) eV, which accurately matches the DFT bands. The edge states for the semi-infinite lattice are shown in Figure 2f. A helical edge state appears on each side of the sample with both spin channels on the right (Figure 2f, right) and left (Figure 2f, left) sides, connecting the valence bulk band with the conduction bulk bands. Figure 2f is a hallmark of QSH insulators, that is, the bulk states connected by topologically nontrivial edge states.

To identify these edge states, we have considered nanoribbon widths of ∼58 nm for (BHT-Ni)_l_ (see Figure S4). The value of Z_2_ = 1 is a direct result of our calculations performed using the WannierTools package. So, (BHT-Ni)_l_ is a QSH insulator or, due to its organic structure, a 2D-OTI. The possibility of using various metal atoms and molecular ligands makes organic topological materials highly tunable, which is one of their advantages.^26^

Two key lattice models derived from the Z_2_ theory provide essential insights for the design of 2D topological insulator materials.^78^ The first model, introduced by Kane and Mele,^79^ demonstrates that SOC opens a band gap at the Dirac point in graphene-like materials, thereby driving the system into a topologically nontrivial phase. The second model, proposed by Bernevig, Hughes, and Zhang,^80^ focuses on SOC-induced band inversion, between valence and conduction bands with opposite parity. In the case of (BHT-Ni)_l_, the topological phase is primarily based on the Kane-Mele model, which emphasizes the role of SOC in the emergence of a nontrivial gap at the Dirac point. The adoption of the GGA method is motivated by the relevance of the Kane-Mele model^81^ in these systems as well as the findings from the previous studies, indicating that the obtained picture is physically meaningful.^50,82^

At this point, it is important to investigate the relationship between SHC and crystal symmetry. The crystal symmetry plays a critical role as it significantly affects the spin–orbit interactions that drive the SHC. The SHC refers to the generation of spin currents perpendicular to an applied electric field in materials with SOC. Understanding this relationship is key as the symmetry properties of a material dictates the allowed forms of SOC in a crystal, which in turn affects the magnitude and even the directionality of SHC. For instance, certain crystal symmetries may enhance or suppress spin–orbit interactions, thereby influencing the efficiency of spin current generation.^83^ To investigate this, we first calculated the SHC of (BHT-Ni)_p_ using MLWF, and later for (BHT-Ni)_l_. Due to the 2D structure of (BHT-Ni)_p_, which confines the charge and spin currents within the *xy* plane,^83^ the *P*6/*mmm* crystal structure of (BHT-Ni)_p_ has only one independent element of SHC (σ_*xy*_^*z*^).^65^

In addition, we will investigate the impact of Fermi level variations by electron doping on the SHC. Figure 3a illustrates the band structure of (BHT-Ni)_p_ including SOC, and Figure 3b depicts the corresponding SHC, σ_*xy*_^*z*^, and its quantization at two nontrivial band gaps (Γ and *K* points) that are not at the Fermi level. The value of the SHC, σ_*xy*_^*z*^, of (BHT-Ni)_p_ at the Fermi level is −0.093 (*ℏ*/e) (S/cm); actually, it is near zero due to the trivial gap combined with both the SIS and TRS of the system. Figure 3a,b shows that moving *E*_F_ to the nontrivial gaps can increase SHC to its maximum values within both SOC gaps. By adding two electrons per unit cell and raising the Fermi level (Figure S5a), the value of SHC reaches its maximum value of −325 (*ℏ*/e) (S/cm) at the SOC gap in (BHT-Ni)_l_ (see Figure S5b). This value is comparable in magnitude to those found in other topological insulators.^67^ As expected, the SHC values in 2D topological insulators should be quantized. In Section A of the Supporting Information file, the calculation of the quantized value of the SHC (in units of $\frac{e}{2\pi }$ ) for (BHT-Ni)_l_ is presented. This quantized value is observed at two nontrivial gaps.

**Figure 3:**
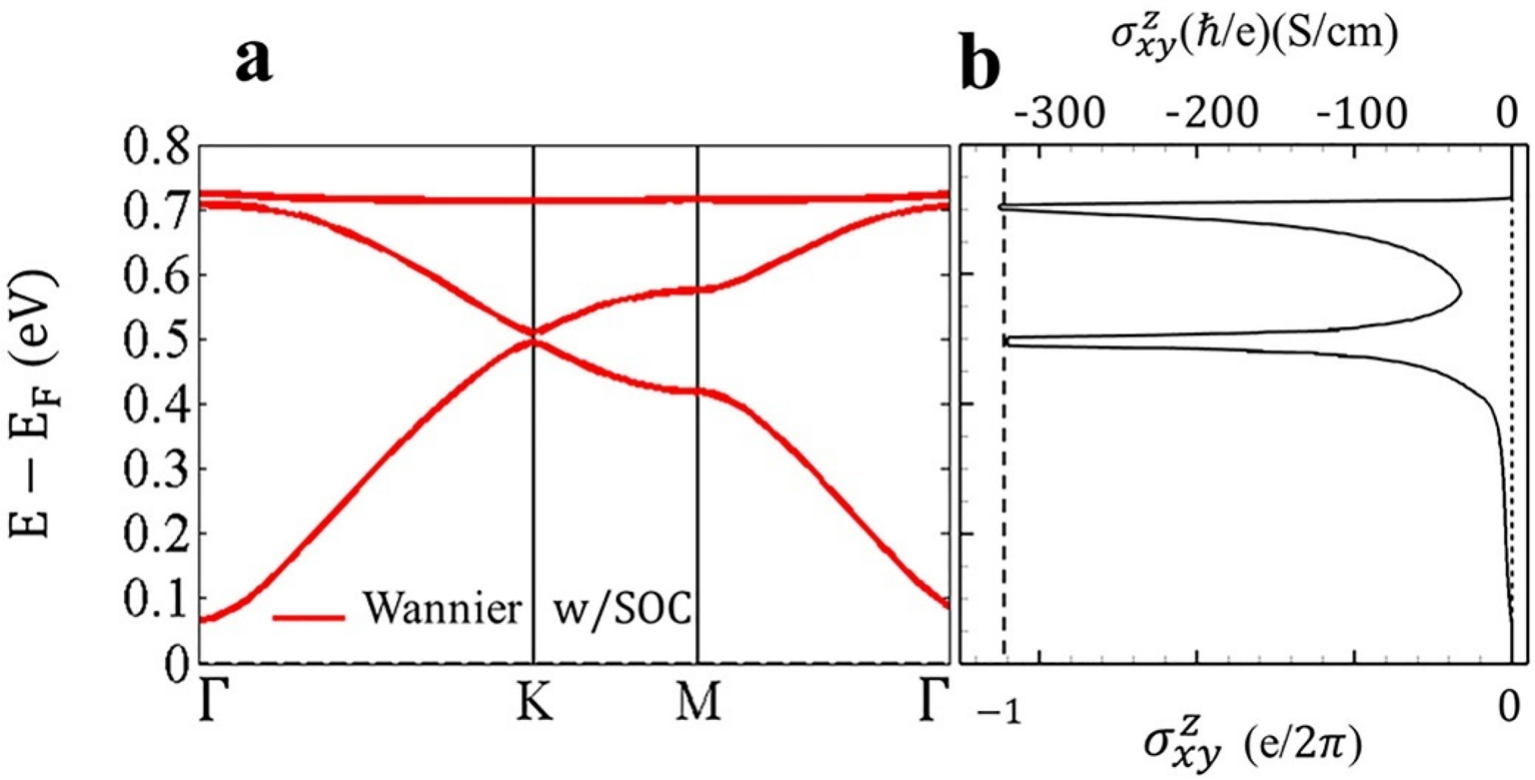
(a) Band structure of (BHT-Ni)_p_ in the Wannier basis by considering SOC. (b) σ_*xy*_^*z*^ tensor element of SHC as a function of the *E*_F_ position for the (BHT-Ni)_p_ sheet, showing that its value at the Fermi level is negligible.

A more comprehensive understanding of the origin of the finite SHC inside the nontrivial gap can be obtained by analyzing the contribution of the bands in the vicinity of the *E*_F_. Figure 4a,b illustrates the band-projected SBC, i.e., Ω_*n*,*xy*_^*z*^(***k***) in eq 4, for energy bands close to the SOC gap in (BHT-Ni)_p_ and (BHT-Ni)_l_, respectively. The color of the bands corresponds to the sign and magnitude of the SBC, i.e., sgn (Ω_*n*,*xy*_^*z*^(***k***)) log |Ω_*n*,*xy*_^*z*^(***k***)|. Figure 4b depicts a large contribution of conduction and valence bands to the SBC, especially in the vicinity of the Fermi energy. Furthermore, the sign of Ω_*n*,*xy*_^*z*^(***k***) changes rapidly as the energy approaches the SOC gap. The significant value of the SBC and its rapid sign change at *E*_F_ suggest that the SHC is related to the topological order of the bands.^67^ We also note that previous calculations on trivially gapped semiconductors^84^ have revealed a nonzero residual SHC within the gap; however, its source does not appear to be topological. Hence, the bulk in TIs could produce a finite spin current even if the Fermi level is situated within the gap. A finite SBC and the sign flip at the Γ point in Figure 4a,b demonstrate its topological nature, which is confirmed by the quantized SHC with a small plateau. This small plateau is due to the smaller SOC gap at the Γ point compared to the SOC gap at the *K* point, as shown in Figure S5b at around 0.2 eV.

**Figure 4:**
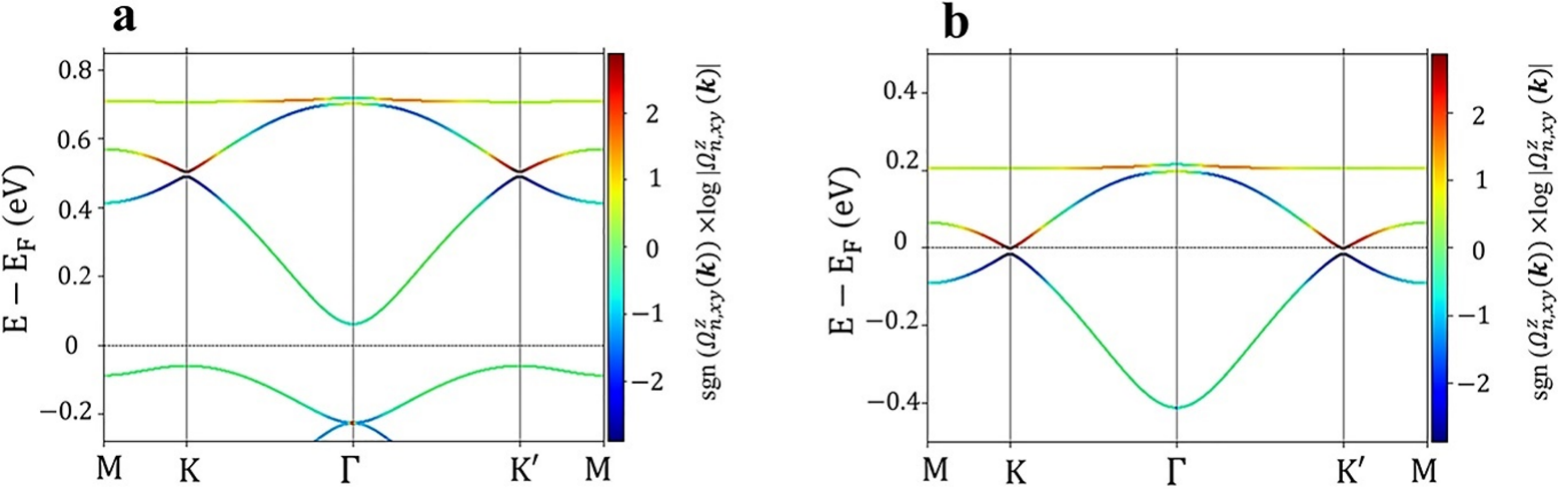
Band-projected SBC, (Ω_*n*,*xy*_^*z*^(***k***)), in the vicinity of *E*_F_ for (a) (BHT-Ni)_p_ and (b) (BHT-Ni)_l_. The energy axis is relative to the Fermi energy, denoted by the gray horizontal line. The color of the bands represents the sign and magnitude of the SBC, i.e., sgn (Ω_*n*,*xy*_^*z*^(***k***)) log |Ω_*n*,*xy*_^*z*^(***k***)|.

We calculated the 2D distribution of the ***k***-resolved SBC in eq 3 at *E* = *E*_F_ for (BHT-Ni)_p_ and (BHT-Ni)_l_. The result for (BHT-Ni)_l_ is shown in Figure 2g. In the case of (BHT-Ni)_p_, the SBC was close to zero, indicating that the SBC is highly sensitive to the position of the Fermi energy, which is consistent with recently reported data.^47^ The red color in the ***k***-resolved SBC denotes positive values.

We have also studied the SHA of (BHT-Ni)_p_ and (BHT-Ni)_l_ structures based on eq 5. To this end, we calculated the EC tensor components (in S/cm), which together with the corresponding SHC and SHA values are reported in Table 2. The calculated SHA of 1.89 is comparable to that exhibited by other TIs, 0.1 < θ_SH_ < 1.0,^67^ the heavy metals such as platinum, with high SHC ∼ 2000 (*ℏ*/e) (S/cm) and 0.056 < θ_SH_ < 0.16,^85^ or tantalum with 0.12 < θ_SH_ < 0.15.^86^ Owing to the finite SHC and confined longitudinal charge conductivity, the SHA of TIs is comparable to those of heavy metals. Therefore, TIs are an ideal choice for energy-efficient charge-to-spin conversion in spin-based devices. As mentioned previously, this implies that for the same spin current, TIs require a lower value of charge current compared to heavy metals, which have a fairly higher conductivity. In Table S2, we summarized the SHA for some metals, semiconductors and TIs.

**Table 2:** SHC of (BHT-Ni)_p_, (BHT-Ni)_l_, *trans*-(BHT-Ni)_p_, and *trans*-(BHT-Ni)_l_ in Units of (ℏ/*e*) (S/cm) at Their *E*_F_, along with the Longitudinal Elements of the EC in Units of (S/cm) at *E*_F_ and the Dimensionless SHA (θ_SH_) for These Four Structures

structure	σ_***xy***_^***z***^	σ_*xx*_	σ_***yy***_	SHA
(BHT-Ni)_p_	–0.093	46.80	46.81	–0.004
(BHT-Ni)_l_	–325.00	343.76	343.88	–1.89
*trans*-(BHT-Ni)_p_	–0.254	5.84	5.85	–0.09
*trans*-(BHT-Ni)_l_	–325.00	332.00	331.92	–1.96

### 3.2 Electronic and Topological Properties in the High Electron Doping Regime of BHT-Ni

Based on the observations of Wang et al.^26^ and Zhang et al.,^28^ hereby we explore the resultant electronic, structural, and magnetic properties that emerge from high electron doping concentration in (BHT-Ni)_p_. Hence, we further increased the electron doping concentration to 2.15 × 10^14^ cm^–2^ (four electrons per unit cell), which is experimentally accessible as a low electron doping concentration level. With this, we aim to provide a detailed understanding of how this doped system behaves, potentially revealing novel properties and eventually filling the knowledge gap left by Wang et al.^26^

Note that at this doping level, the lattice constant is extended only by less than 2%, and hence the (BHT-Ni)_h_ framework remains thermodynamically stable. The electron density difference plots for (BHT-Ni)_l_ and (BHT-Ni)_h_, compared to the pristine structure, are depicted in Figure S6, illustrating the location of the doped charges.

From qualitative arguments, the Fermi level is anticipated to rise into the space between the TFB and the upper Dirac band assuming that the Kagome bands remain unchanged. Nonetheless, our DFT analyses revealed that, at this specific doping level, *E* (***k***, ↑) ≠ *E* (−***k***, ↓), and TRS is broken, resulting in the emergence of spontaneous electron spin polarization with a magnetic moment of 2.0 μ_B_ per unit cell, as estimated from the spin density. Figure S7 illustrates the spin-polarized electron density, obtained from the difference in electron density between spin-up and spin-down channels (δρ = ρ↑ – ρ↓). Each sulfur atom exhibits a magnetic moment of 0.027 μ_B_, while each Ni atom shows a magnetic moment of 0.211 μ_B_, contributing approximately 16.20% and 31.65% to the overall magnetic moment, respectively.

Because of the overlap between the TFB and Dirac bands (see Figure 5a), electrons start to occupy the TFB as the Fermi level is elevated into this region. The instability caused by this leads to spin polarization. Consequently, the spin degeneracy of the Kagome bands is lifted. In this scenario, the Kagome bands of one spin channel (spin-up) become fully occupied by electrons, while only one Dirac band of the other spin channel (spin-down) is filled. The Fermi level aligns precisely with the Weyl point of the spin-down channel, as illustrated in Figure 5b. While spin-polarized Dirac cones have been observed multiple times,^87^ to the best of our knowledge, spin-polarized Dirac cones resulting from electron doping have only been reported by Zhang et al. in electron-doped HTT-Pt.^28^

**Figure 5:**
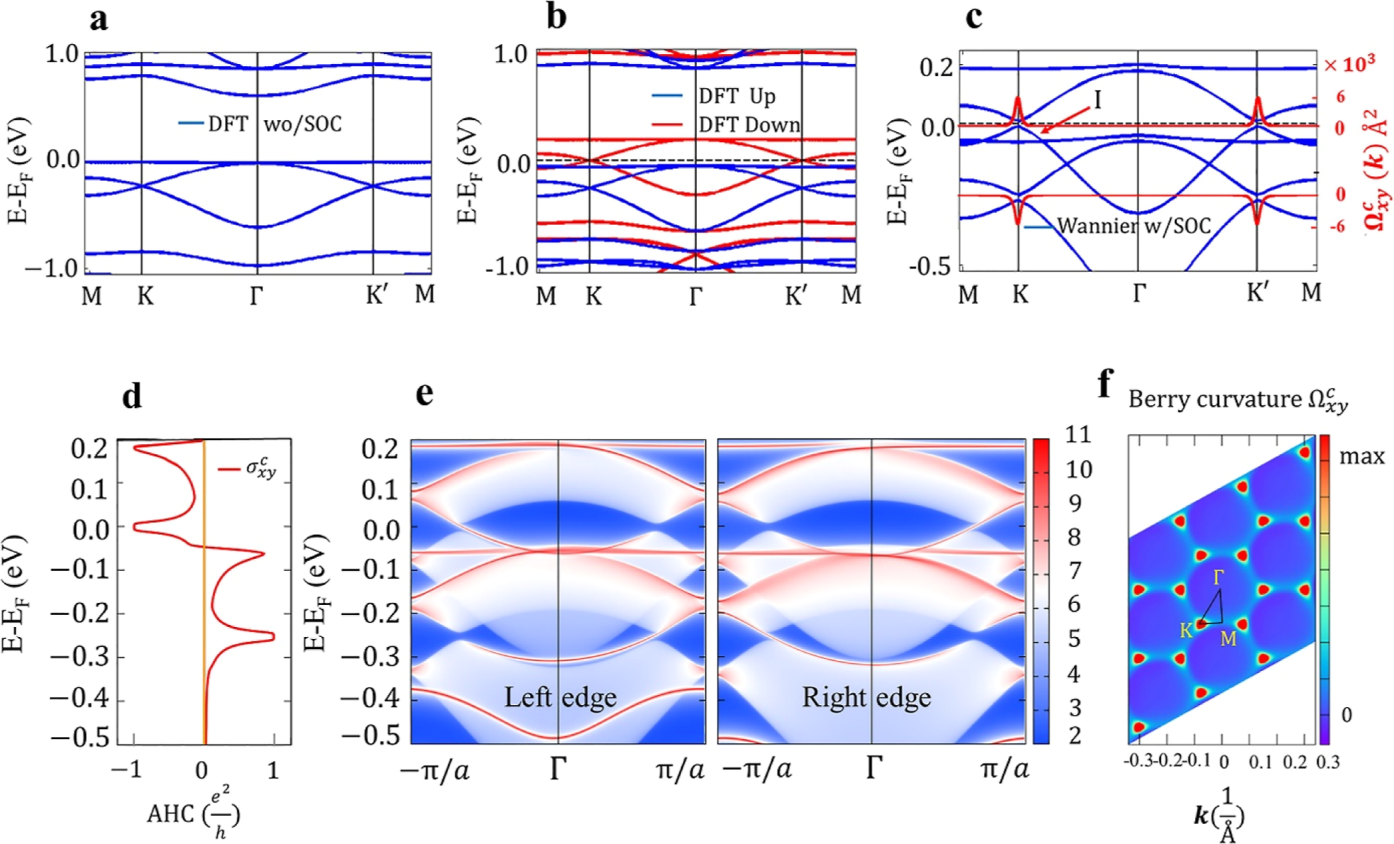
(a) Band structure of (BHT-Ni)_h_ from first principles (DFT) without SOC. (b) Spin-polarized band structure of (BHT-Ni)_h_ calculated without SOC, with spin-down shown in red and spin-up in blue and (c) calculation with Wannier basis sets and SOC; six separated Kagome bands and nontrivial gaps with BCs (red curves) are shown. (d) Calculated quantized AHC. (e) Semi-infinite chiral edge states near the Fermi level based on MLWFs. (f) Distribution of the BC Ω_*xy*_^*c*^ in the 2D momentum space for band I (valence band maximum) marked in (c).

Taking into account SOC and spin polarization, the present DFT computations predict the emergence of an 18 meV band gap at the Fermi level, as shown in Figure 5c. Using MLWFs based on the initial guess (obtained from the projected band structure similar to Figure 2c) in the energy window (*E*_F_ −0.65 eV, *E*_F_ +0.3 eV), the Kagome bands can be accurately approximated using Wannier interpolation, as also depicted in Figure 5c. The heavy electron doped (BHT-Ni)_h_ exhibits topological nontriviality, which can be demonstrated by calculating the Chern number (*C*). Figure 5d illustrates the AHC as a function of the Fermi energy shift. It shows that the AHC forms plateaus in units of e^2^/*h* at the Fermi level, signifying that (BHT-Ni)_h_, behaves as a Chern insulator with a nonzero Chern number (*C* = 1).

The distribution of the ordinary BC, Ω_*xy*_^*c*^, in the 2D momentum space of the lower Dirac band (band I, or valence band maximum, in Figure 5c) is illustrated in Figure 5f. Because of the 2D structure of (BTH-Ni)_h_, the calculated BC has only one nonzero component, Ω_*xy*_^*c*^. One can see that the nonzero BC is mainly localized around the *K* and *K* ^′^ points with the same sign, in agreement with the Chern number calculation. To provide additional validation of the topological nontriviality of (BHT-Ni)_h_, we calculated the edge states of the spin-polarized (BHT-Ni)_h_. The chiral edge states depicted in Figure 5e clearly show the characteristics of TRS breaking as only a single spin channel connects the bulk states (refer to Figure S8 for a zoomed-in view). The calculated nonzero Chern number and the topologically nontrivial edge states in (BHT-Ni)_h_ show great potential for realizing the QAH effect with a single spin-polarized edge channel. Although the QAH phase in TIs has frequently been reported by doping with magnetic atoms^88^ or through the introduction of proximity coupling with antiferromagnetic structures,^89^ here we accomplish this through electron doping. Similar to Zhang et al., who suggested an electron doping approach for inducing ferromagnetism in a TI and the realization of a Chern insulator,^28^ we adapt their method. The Chern number *C* and AHC (σ_*xy*_^*c*^) are related as shown in eq 8 and can be obtained by integrating the BC over the first BZ.

8 $$C=\frac{1}{2\pi }\int_{BZ}d^{2}k\Omega_{xy}^{c}\left(k\right)\;\text{and}{\;\sigma }_{xy}^{c}=-C\frac{e^{2}}{h}$$

The quantization of the calculated AHC (σ_*xy*_^*c*^ = –323 S/cm) in (BHT-Ni)_h_ becomes evident when expressed in units of ( $\frac{e^{2}}{h}$ ) as

$$\sigma_{xy}^{c}\left(S/\text{cm}\right)=-323\frac{S}{\text{cm}}\times 1.2\times 10^{-7}\text{cm}\times \frac{e^{2}/h}{\left(3.874\times 10^{-5}\right)S}=-1\frac{e^{2}}{h}$$

On each side of the sample, a chiral edge channel appears, with opposite direction of propagation on right and left sides (Figure 5e). The *C* = 1 value for the Chern number characterizes a quantized Hall conductivity, which confirms the anticipated topological nontriviality of the SOC gap. As noted, the magnitude of AHC at the Fermi level reaches σ_*xy*_^*c*^ = –323 S/cm or Ω^–1^ cm^–1^, which is comparable to the large AHC magnitude of the Kagome materials.^16^ Hence, by controlling charge DOF, the realization of different topological phases (QSH and QAH) at (BHT-Ni)_p_ is predicted, in which each phase exhibits distinct topological characteristics.

### 3.3 Impact of Geometrical Symmetry Breaking on Topological Behavior of BHT-Ni

In the preceding sections, we have analyzed the effect of charge and spin DOFs on the electronic and topological properties. Here, we focus on the impact of adjusting lattice and orbital DOFs. To this end, by replacing half of the sulfur ligands in (BHT-Ni)_p_ with selenium, we obtain the cis-like and trans-like configurations, hereafter denoted as *cis*-(BHT-Ni)_p_ and *trans*-(BHT-Ni)_p_. The entire real-space structures of *cis*-(BHT-Ni)_p_ and *trans*-(BHT-Ni)_p_ along with their related unit cells are depicted in Figure 6a–d, respectively. Note that the S and Se ligands are coordinated to the central Ni^2+^ ion in cis- and trans-like configurations, with the SIS being broken in the former and preserved in the later. To assess the stability of *cis*-(BHT-Ni)_p_ and *trans*-(BHT-Ni)_p_, we rely on the calculated cohesive energies (E_C_ as defined in eq 1), which are reported in Table 1. Both configurations exhibit good stability when compared to graphene. Furthermore, we examine the electronic structure of reduced symmetry configurations for both cis- and trans-like arrangements in their pristine and low electron doping counterparts. In *trans*-(BHT-Ni)_p_, the crystal space group is *P*6/*m* (no. 175), with the reduced point group C_6h_ in Schönflies notation, which is a subgroup of D_6h_. In this case, some mirror symmetries are broken, but the SIS is retained. In contrast, in *cis*-(BHT-Ni)_p_ with the elimination of SIS, the crystal point group is reduced to D_3h_ in Schönflies notation, and its space group corresponds to *P*6̅2*m* (no. 189).

**Figure 6:**
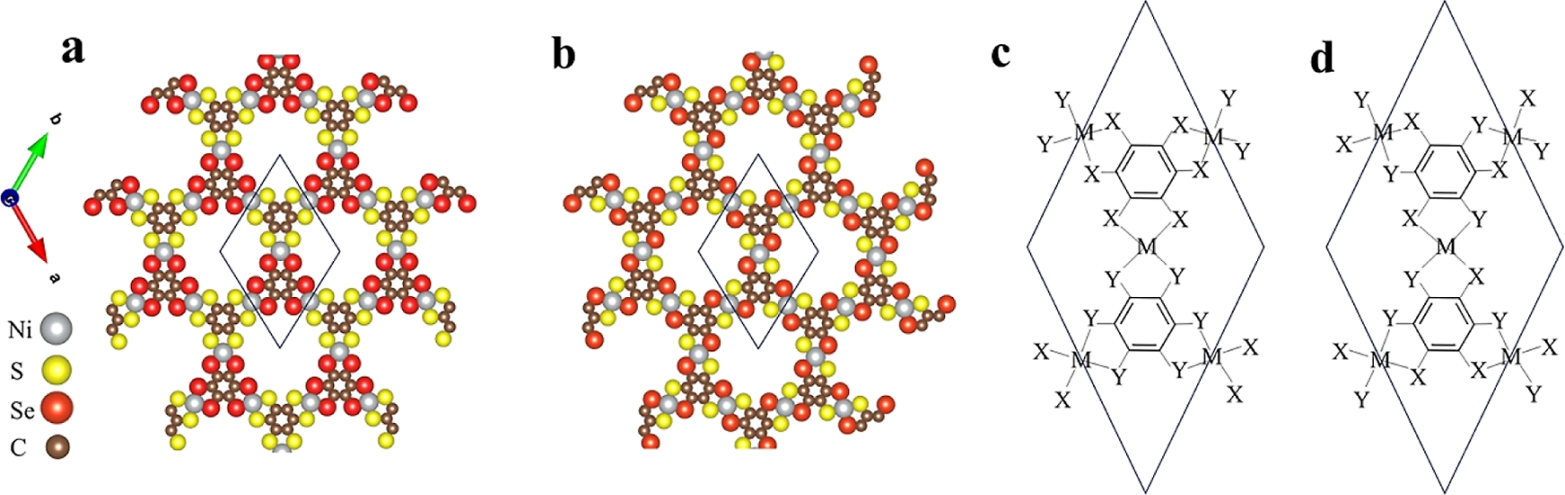
Molecular structure of *cis*- and *trans*-(BHT-Ni)_p_. (a,b) Top view of *cis*-(BHT-Ni)_p_ and *trans*-(BHT-Ni)_p_, with gray, yellow, red, and brown circles representing Ni, S, Se, and C atoms, respectively. The solid line indicates the unit cell. (c) Structure of the unit cell [solid line in (a)] of the proposed MOFs (M = Ni; X = S, Y = Se) for the cis-like configuration and (d) for the trans-like configuration.

Remarkably, the analysis of the PDOS for each element in the *trans*-(BHT-Ni)_p_ and *cis*-(BHT-Ni)_p_ structures, compared to the (BHT-Ni)_p_, clearly shows that the Kagome bands of (BHT-Ni)_p_ near the Fermi level are dominated by the S p_*z*_ orbitals and Ni d_*xz*_ and d_*yz*_ orbitals, with a minor contribution from C p_*z*_ orbitals. However, there is a more significant contribution near the Fermi level from Se p_*z*_ orbitals compared to that of sulfur atoms for cis-like and trans-like structures. See Figure S9a–c for the PDOS of the (BHT-Ni)_p_, *trans*-(BHT-Ni)_p_, and *cis*-(BHT-Ni)_p_, respectively.

Figure S10 shows the band structure for both cis- and trans-like structures without including SOC. Both *cis*-(BHT-Ni)_p_ and *trans*-(BHT-Ni)_p_ retain their semiconductor nature, and the Dirac point is preserved in *trans*-(BHT-Ni)_p_ (Figure S10a). However, breaking of the SIS in *cis*-(BHT-Ni)_p_ leads to a gap opening of 64 meV at the Dirac point (see Figure S10b). Before accounting for SOC effects, the bands are spin-degenerate as (BHT-Ni)_p_, consistent with the diamagnetic nature of the square planar complexes with a d^8^ electron configuration. The SOC gaps for the cis- and trans-like structures are reported in Table S3 [see Figure S10c for the band structure of the *trans*-(BHT-Ni)_p_, including SOC].

Two distinct band gaps emerge in the cis-like configuration: one resulting from SIS breaking (Figure S10b) and the other arising due to SOC in the presence of SIS breaking (Figure S10d). Apart from the gap openings induced by SOC, by comparing the band gaps of the trans-like structure with those of the (BHT-Ni)_p_, it become evident that the nontrivial Dirac gap in *trans*-(BHT-Ni)_p_ is relatively small (14 vs 12.6 meV). One possible explanation could be the existence of hybridization between the Ni d-orbitals and the p-orbitals of light atoms (C, S, and Se atoms) that affects the strength of SOC.^90^ As noted earlier for the *trans*-(BHT-Ni)_p_, the Kagome bands display a larger contribution from the p_*z*_ orbital of Se atoms compared to those of S atoms. Consequently, the larger atomic radius of Se atoms compared to that of S atoms leads to an increase in hybridization and a decrease in the SOC Dirac gap. For a comparison of the SOC gaps of the low electron doping counterparts of (BHT-Ni)_p_ and *trans*-(BHT-Ni)_p_, see Table S3.

In the cis-like configuration with broken SIS and including SOC, Zeeman-type spin splitting of the energy bands is observed at all ***k***-points in the BZ, except for special points like M and Γ (see Figure S10d). Although the spin-up and spin-down bands are no longer separable upon including SOC, the z component of the spin ( ${ŝ}_{z}$ ) is approximately a conserved quantum number close to the *K* and *K* ^′^ points. Therefore, using the projection of spin operator ${ŝ}_{z}$ —i.e., $\left\langle \psi_{\text{nk}}\left|{ŝ}_{z}\right|\psi_{\text{nk}}\right\rangle$ —obtained from Wannier interpolation, we derived the ${ŝ}_{z}$ projected band structure of *cis*-(BHT-Ni)_l_, as shown in Figure 7a. In general, under SOC, SIS breaking removes the spin degeneracy of the energy bands at two valleys, *K* and *K* ^′^. Due to TRS, the spin splitting in opposite valleys must be reversed, as shown in Figure 7b; hence, the spin moments can also be used to identify the valley carriers. This forms the foundation of the coupled spin and valley physics. In this respect, there is a direct band gap at the two inequivalent corners, *K* and *K* ^′^, of BZ. It should be notice that the spin splitting occurs in both the valence band maximum and the conduction band minimum of *cis*-(BHT-Ni)_l_ (see Figure 7b), unlike the spin splitting in MoS_2_,^91^ where only in the valence band maximum spin splitting occurs. Since the conduction band minimum at MoS_2_ consists of the Mo (d_*z*_^2^) orbital, SOC is inactive, and the conduction band minimum remains spin degenerate. The magnitude of spin splitting depends on the relative strength of SOC in the materials.

**Figure 7:**
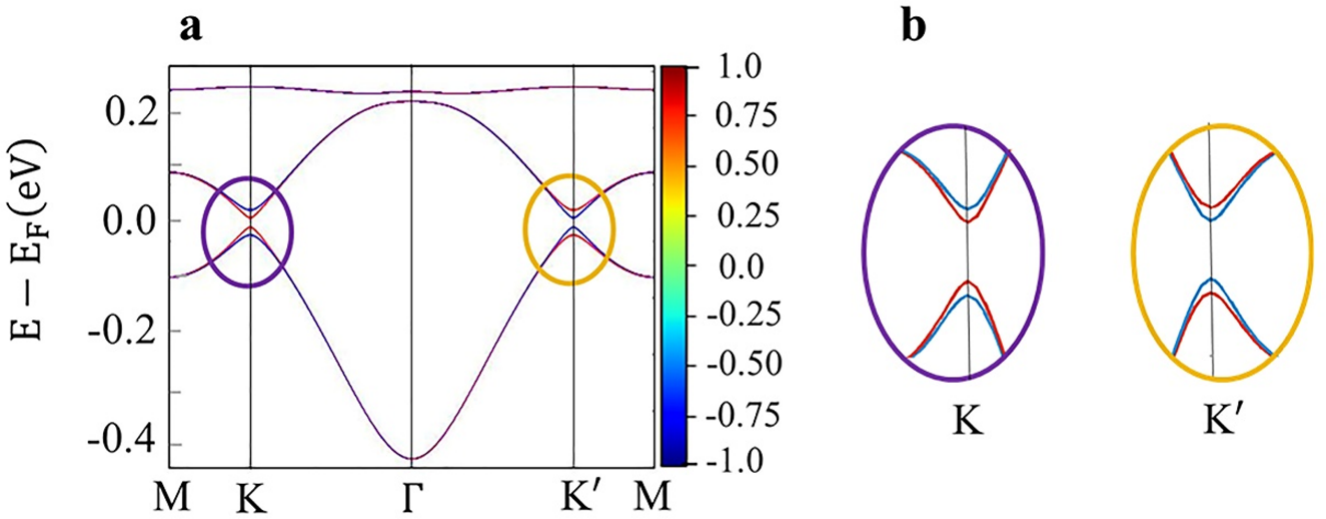
(a) Fully relativistic band structure of *cis*-(BHT-Ni)_l_ and the projection of the *z*-component of spin operator ${ŝ}_{z}$ (color map). The red and blue colors indicate spin-up and -down states, respectively. The color scheme represents the expectation value $\left\langle {ŝ}_{z}\right\rangle$ of the spin operator ${ŝ}_{z}$ in units of *ℏ*/2. (b) Magnification of the regions specified in (a).

Next, we analyze the relevant topological properties and, in particular, consider the intrinsic contribution of the SHC, which is independent of any scattering. As (BHT-Ni)_l_, *trans*-(BHT-Ni)_l_ also exhibits a large SHC by moving the chemical potential into the SOC Dirac gap (see Figure S11a,b). The calculated SHC (σ_*xy*_^*z*^) = −325 (*ℏ*/*e*) (S/cm) for *trans*-(BHT-Ni)_l_ indicates the preservation of quantization (see Figure S11b). To determine the SHA, we calculated the longitudinal EC, σ_*yy*_, by using the Boltzmann transport equation. It is important to mention that the shift in *E*_F_ results in a change in electron concentration in the calculation of EC. Different elements of EC and the SHA of *trans*-(BHT-Ni)_p_ and *trans*-(BHT-Ni)_l_ are reported in Table 2. A comparison of the reported data on the SHA in Table 2 reveals a higher SHA in *trans*-(BHT-Ni)_l_ compared to that in (BHT-Ni)_l_. In addition to the quantized SHC, the edge states connecting the bulk states are another reason for maintaining the QSH phase in the *trans*-(BHT-Ni)_l_ (see Figure S12).

Although the breaking of SIS in *cis*-(BHT-Ni)_l_ leads to the disappearance of the SHC, another topological characteristic, BC, emerges. In the *cis*-(BHT-Ni)_l_ structure, the charge carriers acquire a valley-contrasting BC.^92^ The BC, Ω_*n*,*xy*_^*c*^(***k***), of the occupied states can be expressed as shown in eq 4. As outlined by Vanderbilt,^93^ when a crystal possesses both SIS and TRS, the BC is zero. However, upon breaking SIS in *cis*-(BHT-Ni)_l_, a nonzero BC emerges (see Figure 8a) similar to the effect observed with TRS breaking in (BHT-Ni)_h_ (see Figure 5f).

**Figure 8:**
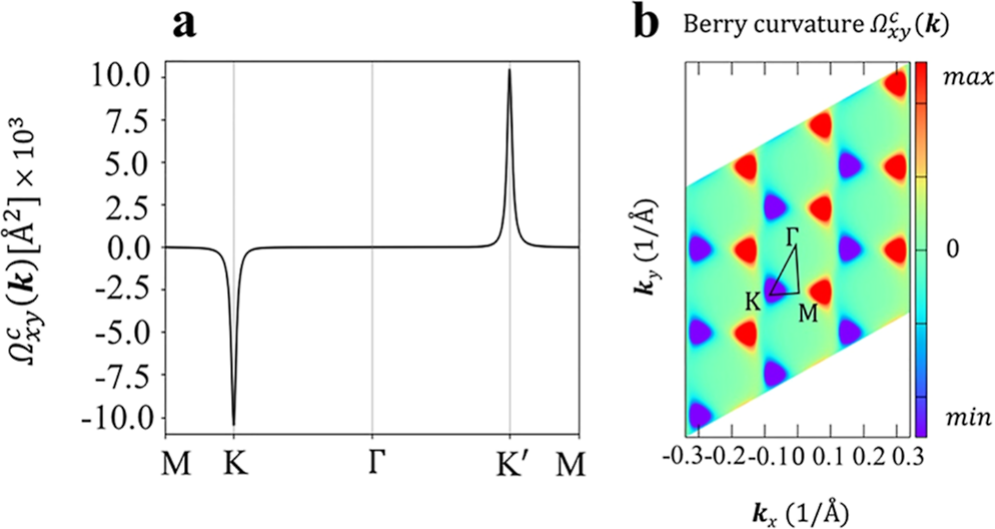
(a) Ordinary BC of *cis*-(BHT-Ni)_l_ with considering SOC, along the high-symmetry lines. (b) Calculated BC distribution of the valence bands below the Fermi level in the (***k***_*x*_, ***k***_*y*_) plane, presented in arbitrary units.

The BC, Ω_*xy*_^*c*^(***k***), of *cis*-(BHT-Ni)_l_ was significantly peaked at both *K* and *K* ^′^ (Figure 8a) but with opposite signs (Ω_*xy*_^*c*^(*K*) = −Ω_*xy*_^*c*^(*K* ^′^)).^92^ The ***k***-space contrasting BC in systems without SIS is a key quantity for characterizing the chirality of the Bloch electrons and serves as the basis for valley-contrasting phenomena like VHE.^92,94^ Away from the two valleys, Ω_*xy*_^*c*^ decays rapidly and vanishes at the Γ and M points (see Figure 8b). According to eq 4, there exists reverse correlation between the gap value and the BC magnitude. So, a small band gap (see Table S3) in *cis*-(BHT-Ni)_l_ has resulted in a large BC in the presence of SOC (10 × 10^3^ Å^2^), namely, the smaller the SOC gaps, the larger the resulting BC. Interestingly, the magnitude of the BC shown in Figure S13a is no longer zero in the absence of SOC. The distribution of the BC in the 2D momentum space of the valence band maximum is illustrated in Figure S13b. Therefore, a VHE is triggered in the planar *cis*-(BHT-Ni)_l_ structure even without accounting for SOC. This behavior differs from that observed in some transition metal dichalcogenides, where SOC is considered as an essential factor in producing the VHE.^95^ Breaking SIS results in two asymmetric sides in the cis-like configuration. The calculated edge states for these two asymmetric sides of *cis*-(BHT-Ni)_l_ indicate the emergence of the VHE (see Figure S12c,d). The magnitude of the BC without including SOC (see Figure S13a) is smaller compared to the one with including SOC (see Figure 8a). This phenomenon arises because, as highlighted earlier, the inclusion of SOC removes spin degeneracy at special points (*K* and *K* ^′^ in Figure 7a). This results in smaller band gaps near the Fermi level (*E*_F_). Consequently, the peaks of the BC with SOC are higher compared to those without SOC.

## 4 Conclusions

The electronic and topological properties of several BHT-Ni materials have been studied, and the influence of lattice-, charge-, spin-, orbital-, and valley DOFs has been assessed. Manipulating the charge relocates the Fermi level to the SOC gaps to achieve the desired topological properties. This requires doping with electrons or holes depending on the topology of the band structure in the material. In the case of (BHT-Ni)_p_, it has been reported that two (or four) electrons per unit cell, corresponding to an electron doping concentration of up to ∼2 × 10^14^ cm^–2^, are needed to shift the Fermi level into the SOC gaps.^26^ To evaluate this, we investigated the topological aspects of electron doping at different concentrations. The QSH state appeared at a low electron doping concentration (1.07 × 10^14^ cm^–2^), where the calculated helical edge states and quantized SHC, in addition to the nonzero Z_2_ topological invariant, overall confirm the QSH state in (BHT-Ni)_l_. The analysis of the SHC in 2D (BHT-Ni)_l_ indicates that σ_*xy*_^*z*^ reaches its maximum value of −325 (ℏ/e) (S/cm). A large SHA is also observed in its low electron-doped counterpart, (BHT-Ni)_l_.

Further increasing the electron doping concentration affects the magnetic properties. Thus, with four electrons per unit cell, four spin-polarized Kagome bands are filled, causing the Fermi level to shift into the Dirac point of the spin-down channel, which leads to the breaking of TRS, (E (***k***, ↑) ≠ E (−***k***, ↓)). This results in a large BC emerging at both *K* and *K* ^′^ points with peaks of the same sign at the Fermi level for both, while the BC in the spin-up channel is obtained with the opposite sign. The calculated chiral edge states, quantized AHC, and the integer Chern number collectively validate the presence of a Chern insulating state in (BHT-Ni)_h_.

Tuning the DOFs enables the engineering of the BC, a key topological property. Initially, by adding two electrons, an SBC was created. Subsequently, increasing the electron doping concentration to four electrons, the SBC transformed into a BC with the same sign at all BZ corners. This occurs due to the SIS, which implies Ω­(***k***) = Ω­(−***k***). Finally, by breaking the SIS in *cis*-(BHT-Ni)_l_ and altering the lattice and orbital DOFs, the BC changes its sign at *K* ^′^. The change in the sign of BC is attributed to the presence of TRS that implies Ω­(***k***) = −Ω­(−***k***). Furthermore, the spin splitting of bands is another exotic property that appears by adjusting the lattice and orbital DOFs, that is nonuniform throughout the ***k*** space. It peaks at the *K* and *K* ^′^ points and diminishes away from them. Finally, modifying the lattice and orbital DOFs in the *trans*-(BHT-Ni)_l_ configuration leads to an enhanced SHA and preserved nontrivial topological properties.

Summing up, the already synthesized trivial (BHT-Ni)_p_ shows a transition between different trivial and nontrivial topological states by altering different DOFs. In this transition, the QSH state appears at a low electron doping concentration (1.07 × 10^14^ cm^–2^) and the QAH state at high (2.15 × 10^14^ cm^–2^) electron doping levels. At first, the QSH state is characterized by a nonzero Z_2_ topological invariant. Subsequently, TRS breaking leads to a robust QAH state with a nonzero Chern number. With simultaneous realization of these phases, different types of topological conductivities corresponding to these phases were also observed. The quantized SHC and AHC, corresponding to (BHT-Ni)_l_ and (BHT-Ni)_h_, respectively, confirm their topologically nontrivial states. Although the SHC of (BHT-Ni)_l_ is small compared to that of heavy metals, its small EC results in a large SHA, similar to those of other TIs. Our theoretical investigations of (BHT-Ni)_l_ not only reveal the interplay between the intrinsic SHC and band topology but also offer a promising material foundation for the future development of spintronic devices. Table 3 summarizes the results of this study.

**Table 3:**
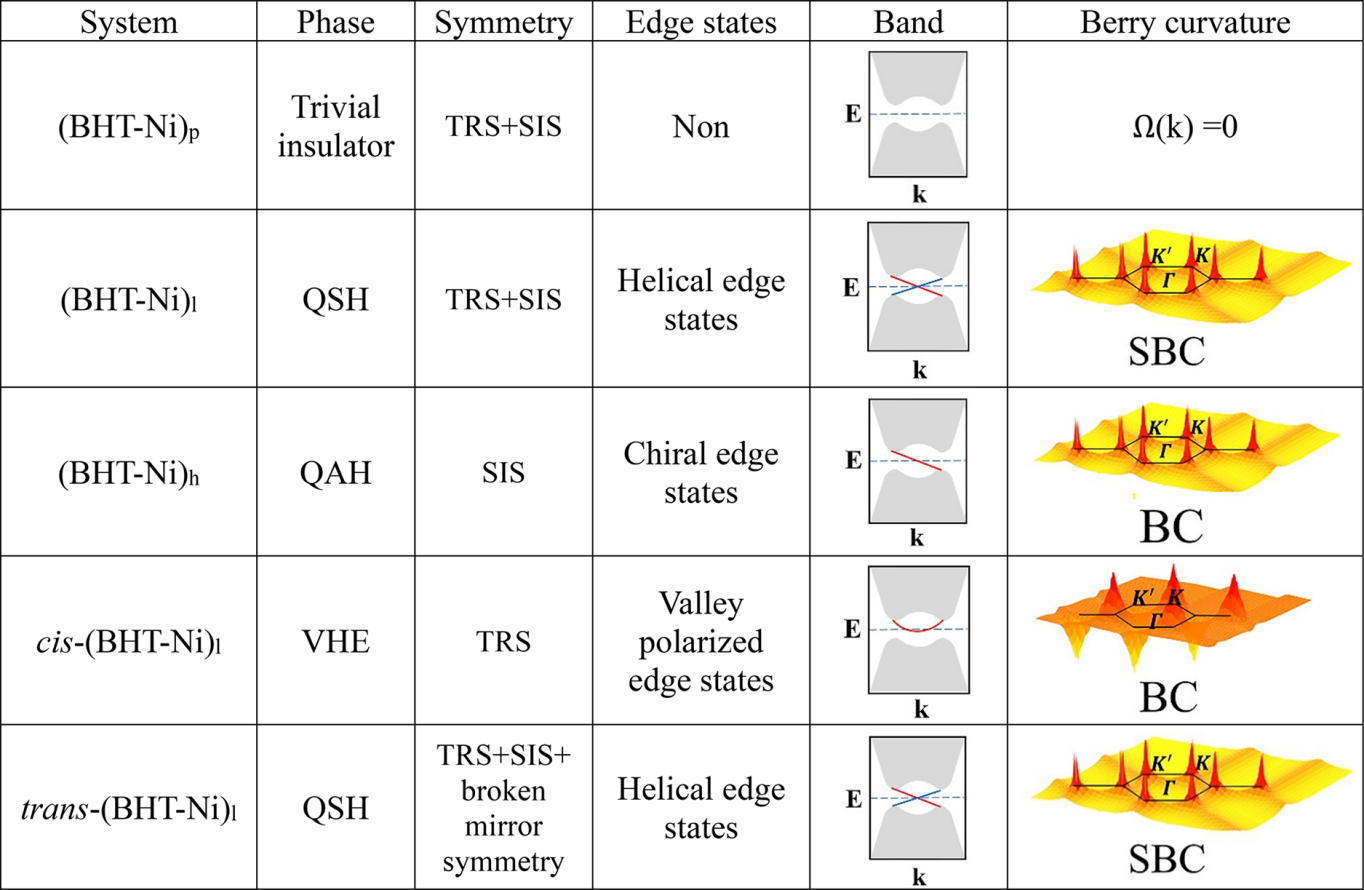
Quick Overview of All Findings from This Study, Illustrating the New Phases—whether Trivial or Nontrivial Topological Phases—That Emerge from Altering Each DOF

## Supplementary Material



## Notes

Additional information including band structures with SOC and Hubbard U term, SHC data for various materials, electron density difference for (BHT-Ni)_h_ and (BHT-Ni)_l_, semi-infinite band structures of *trans*- and *cis*-(BHT-Ni)_l_, and ordinary BC of *cis*-(BHT-Ni)_l_ without SOC (PDF)
