# Supplementary material for: Tuning Topologically Nontrivial States in the BHT-Ni Metal–Organic Framework
Source: J Phys Chem C Nanomater Interfaces. 2025 Jan 27;129(5):2556–69. doi: 10.1021/acs.jpcc.4c06013 (PMC12164724; doi:10.1021/acs.jpcc.4c06013)
Supplement: Supplementary file 1 [file jp4c06013_si_001.pdf]

Supporting Information:

Tuning Topologically Nontrivial States in the BHT-Ni Metal-Organic Framework

Nafiseh Falsafi,[1] Saeed H. Abedinpour[2], Fariba Nazari*,[1,3] Francesc Illas*[4]

[1]*Department of Chemistry, Institute for Advanced Studies in Basic Sciences, Zanjan 45137-66731, Iran*

[2]*Department of Physics, Institute for Advanced Studies in Basic Sciences, Zanjan 45137-66731, Iran*

[3]*Center of Climate Change and Global Warming, Institute for Advanced Studies in Basic Sciences, Zanjan 45137-66731, Iran*

[4]*Departament de Ciència de Materials i Química Física & Institut de Química Teòrica i Computacional (IQTCUB), Universitat de Barcelona,C/Martí i Franquès 1, 08028 Barcelona, Spain*

Corresponding authors: nazari@iasbs.ac.ir, francesc.illas@ub.edu

CONTENTS:

- **Figure S1**: Band structure of the $(BHT\text{-}Ni)_p$ with different magnitudes of the Hubbard U term.
- **Figure S2**: Zoom-in on the band structure of the $(BHT\text{-}Ni)_p$ and $(BHT\text{-}Ni)_l$, including SOC, in the vicinity of the Kagome bands.
- **Figure S3**: Orbital-resolved band structure of the $(BHT\text{-}Ni)_p$.
- **Figure S4**: Band structure of the $(BHT\text{-}Ni)_l$ nanoribbons with different widths.
- **Figure S5**: Band structure of the $(BHT\text{-}Ni)_l$ with quantized SHC at two SOC gaps.
- **A**: Calculation of the quantized value of SHC for the $(BHT\text{-}Ni)_l$.

- **Table S1**: Conventional components of the SHC tensor in all 230 space groups.
- **Table S2**: Comparison of SHC, EC and SHA in various materials from the literature.
- **Figure S6**: Electron density difference of the $(BHT\text{-}Ni)_l$ and $(BHT\text{-}Ni)_h$ compared to the pristine structure.
- **Figure S7**: Spin-polarized electron density difference ($\delta\rho$) of the $(BHT\text{-}Ni)_h$.
- **Figure S8**: Zoom-in on semi-infinite chiral edge states of the $(BHT\text{-}Ni)_h$.
- **Figure S9**: Projected density of states of $(BHT\text{-}Ni)_p$, *trans*-$(BHT\text{-}Ni)_p$ and *cis*-$(BHT\text{-}Ni)_p$.
- **Figure S10**: Band structure of the *cis*- and *trans*-$(BHT\text{-}Ni)_p$, with and without considering SOC.
- **Figure S11**: Band structure of the *trans*-$(BHT\text{-}Ni)_l$ with SOC and the corresponding SHC as a function of the Fermi level position.
- **Table S3**: Comparison of SOC gaps in $(BHT\text{-}Ni)_p$, *cis*- and *trans*-$(BHT\text{-}Ni)_p$ and their charged counterparts.
- **Figure S12**: Semi-infinite edge states of the *trans*-$(BHT\text{-}Ni)_l$ and *cis*-$(BHT\text{-}Ni)_l$.
- **Figure S13**: Ordinary Berry curvature (BC) of the *cis*-$(BHT\text{-}Ni)_l$, without considering SOC.

Figure S1. Band structure of $(BHT\text{-}Ni)_p$ with different magnitudes of the Hubbard U term, including U = 0, 3, and 5. As shown, the gap and the target Kagome bands are not significantly altered by the inclusion of the U parameter, indicating that the choice of U does not affect the overall properties of the target Kagome bands in this case.

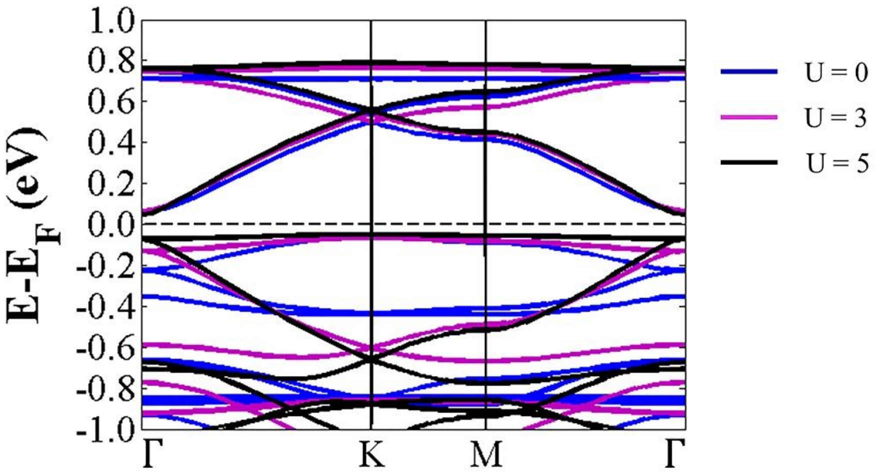

Figure S2. (a) Band structure of $(BHT\text{-}Ni)_p$ showing three SOC gaps. (b) Zoom-in on Figure 2d for band structure of $(BHT\text{-}Ni)_l$, which also shows three SOC gaps.

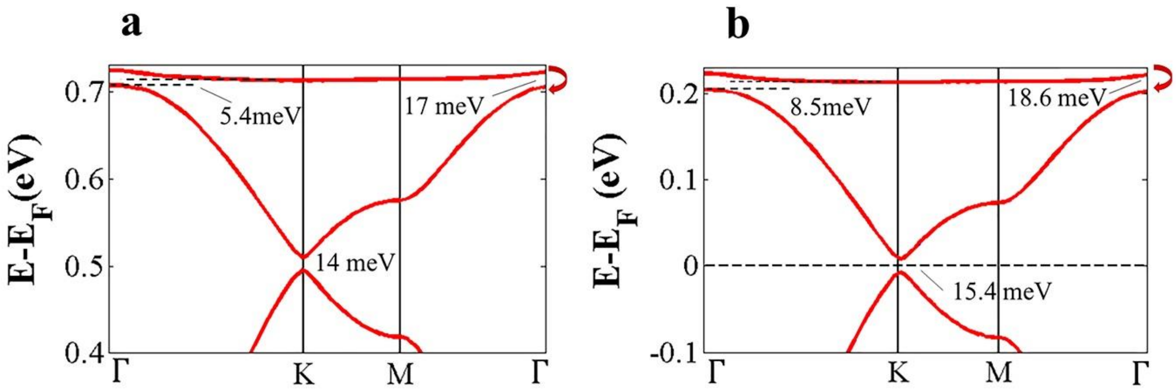

Figure S3. (a) Band structure of the $(BHT\text{-}Ni)_p$. (b) Orbital-resolved (projected) band structure of the Kagome bands on Ni, S and C atoms, showing the contribution of each atom to the Kagome bands.

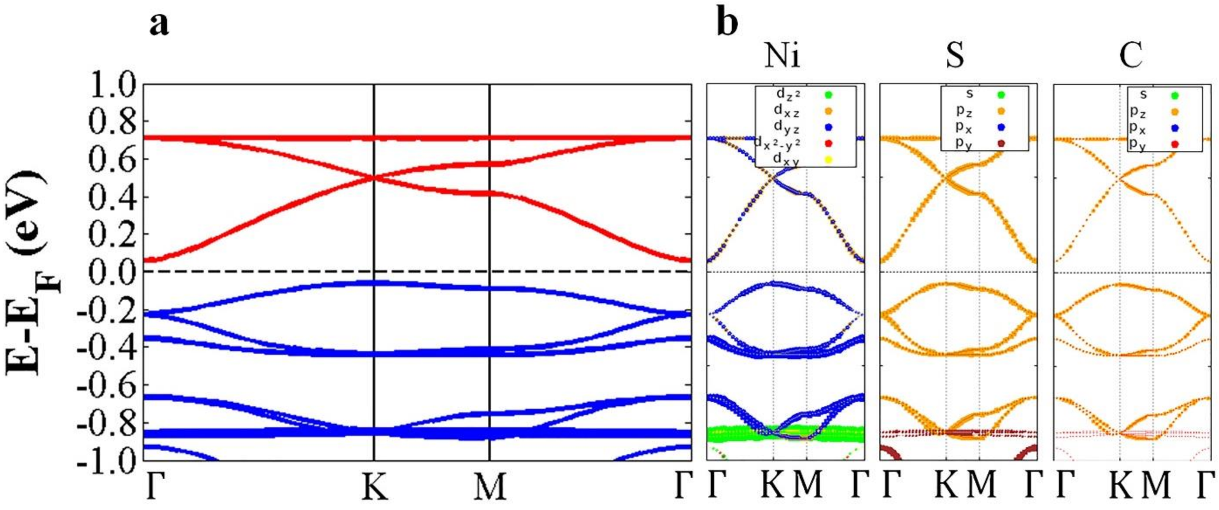

Figure S4. Band structure of (BHT-Ni)$_l$ nanoribbons with widths ranging from two to forty times (~58 nm) their lattice parameter.

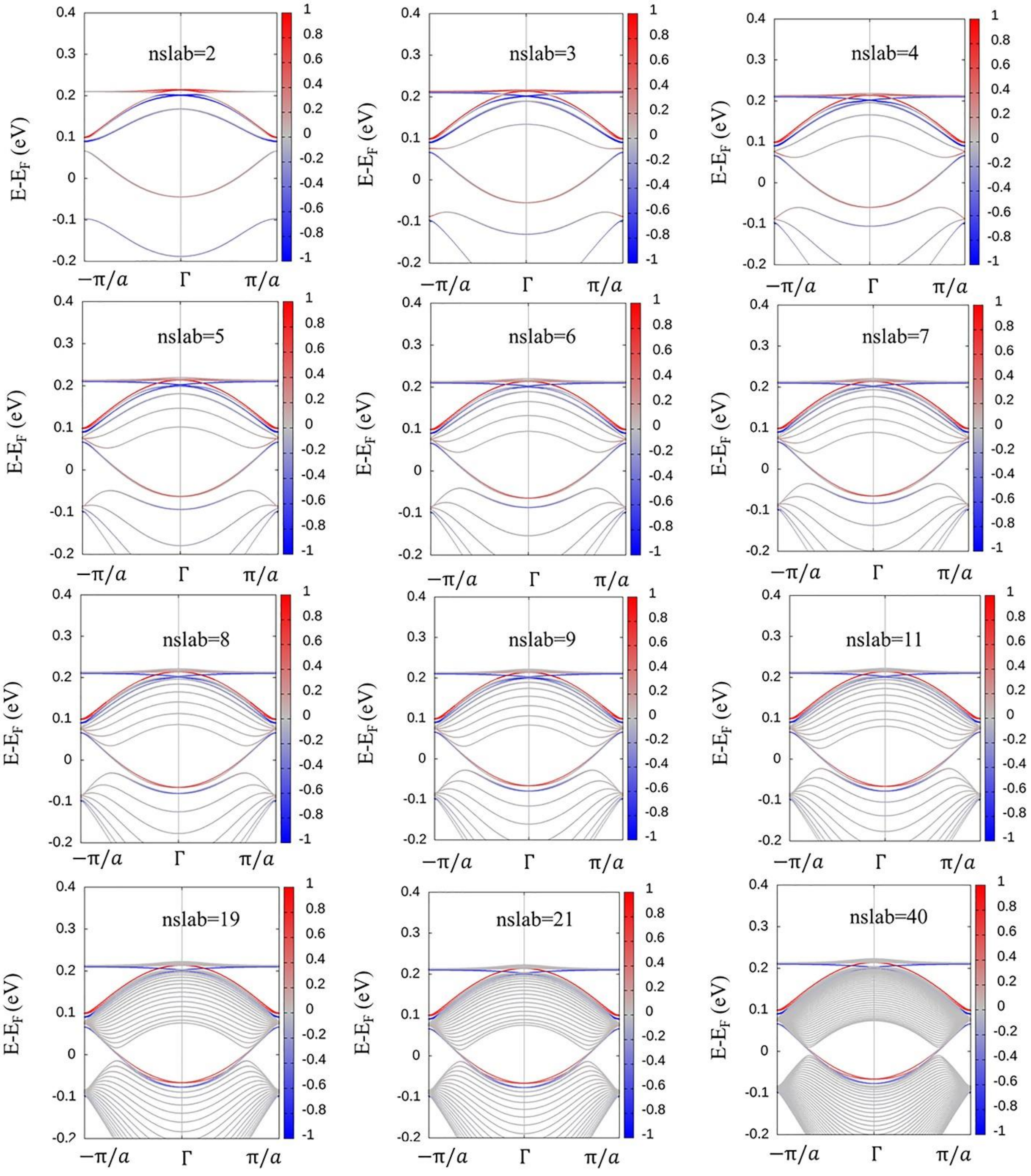

Figure S5. (a) Band structure of $(BHT\text{-}Ni)_l$ in the Wannier basis, with considering SOC. (b) The σ_{xy}^{z} tensor element of SHC as a function of the E_F position for the $(BHT\text{-}Ni)_l$ sheet, illustrating its quantized value at two SOC gaps.

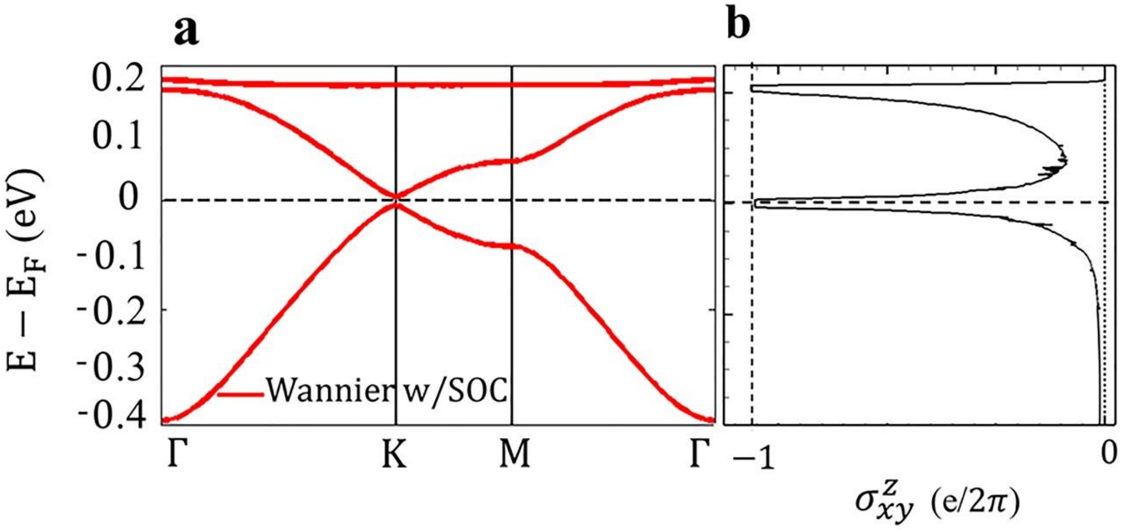

A: Quantized value of SHC

For 2D systems with $\hat{s}_z$ conservation, the spin Chern (C_s) number can be calculated, which is directly related to the SHC (σ_{xy}^{z}) by [1]

$$\sigma_{xy}^{z} = \frac{e}{2\pi}\, C_s$$

$$\sigma_{xy}^{z}\ (\hbar/e)(\mathrm{S/cm}) = -325\ (\frac{\hbar}{e}\frac{\mathrm{S}}{\mathrm{cm}}) \times 1.2 \times 10^{-7}\mathrm{cm} \times \frac{e^2/h}{(3.874 \times 10^{-5})\ \mathrm{S}} = -1\frac{e}{2\pi}$$

The lattice parameter of the $(BHT\text{-}Ni)_l$ unit cell along the z-axis is 12 Å.

Table S1. Conventional components of the SHC tensor categorized by the number of independent components present in all 230 space groups.

Independent components	6	3	2	1
Space group	1-74	75-194	195-206	207-230

Table S2. SHC tensor elements at E_F (T = 0 K) in units of $(\hbar/e)$ (S/cm), electrical conductivity (EC) σ in units of (S/cm) or $\Omega^{-1}\mathrm{cm}^{-1}$, and dimensionless spin Hall angle (SHA) from the literature for some metals, semiconductors and TIs.

Structure	θ_e	σ (S/cm)	σ_{SH}(exp)	σ^x_{yz}	σ^y_{zx}	σ^z_{xy}	θ_t	Ref.
β-Ta	0.15	5.3×10^3		0.8×10^3				[2]
β-W	0.4	4.7×10^3		1.9×10^3				[3]
Pt	0.08	4.2×10^4		3.4×10^3				[4]
Pt	0.068	5×10^4	1.9×10^3					[5]
Pt						2.05×10^3		[6]
Pt				2.21×10^3				a
Pt				2.281×10^3				[7]
Bi_2Se_3	2–3.5	5.7×10^2	2.2–4.0 × 10^3	2.94×10^2			0.26	c[8][9]
$Bi_{0.9}Sb_{0.1}$	52	2.5×10^5		1.3×10^7				[10]
$(Bi_{0.5}Sb_{0.5})_2Te_3$	180-425	2.2×10^2		3.54×10^2			0.79	c[8][11]
Bi_2Te_3	1.0	3.7×10^3	8.0×10^3	4.36×10^2			0.06	c[8][12]
$(Bi_{1-x}Sb_x)_2Se_3$	160	2.4×10^2		2.18×10^2			0.45	[13]
Sb_2Se_3				18.76×10^1				[8]
Sb_2Te_3				2.26×10^2				[8]
$ThAl_2$		0.9×10^4		-8.71×10^2		-9.68×10^2	0.24	[14]
$ThGa_2$		1.7×10^4		-9.03×10^2		-6.05×10^2	0.16	[14]
HfB_2		6.6×10^4		-5.12×10^2		-5.22×10^2	0.02	[14]
ZrB_2		2.5×10^4		-1.94×10^2		-2.02×10^2	0.017	[14]
LaAlGe	0.06	1.25×10^4		-2.57×10^2	-2.14×10^2	-3.51×10^2		[6]
LaAlSi	0.04	1.72×10^4		-2.35×10^2	-2.02×10^2	-3.36×10^2		[6]
WTe_2	0.09–0.5	$(1.4-1.7) \times 10^3$	(0.20-3.0) × 10^2					[15]
WTe_2	0.029	2.6×10^3	0.40 × 10^2					[16]
WTe_2				-0.35×10^2		-2.47×10^2		[6]
WTe_2		1.13×10^3		0.14×10^2	0.96×10^2			[17]
WTe_2				-0.44×10^2	1.03×10^2	-2.04×10^2		[18]
$MoTe_2$	0.032	1.8×10^3	29					[19]
$MoTe_2$				-0.18×10^2	2.86×10^2	-1.76×10^2		[18]
TaAs				-3.57×10^2	-3.82×10^2	-7.81×10^2		[20]
TaP				-3.44×10^2	-4.37×10^2	-6.03×10^2		[20]
NbAs				-2.60×10^2	-3.12×10^2	-3.30×10^2		[20]
NbP				-1.35×10^2	-0.83×10^2	-0.07×10^2		[20]

[a] This work with DFT, ***k***-point 6×6×6, Berry ***k***-mesh 100×100×100, adaptive refinement ***k***-mesh of 4 × 4 × 4 and [c] means calculation, e in θ_e means experimental, and t in θ_t means theory.

Figure S6. (a) Top view and (b) side view of the electron density difference for low electron doping concentration, $(BHT\text{-}Ni)_l$, compared to the pristine structure. (c) Top view and (d) side view of the electron density difference for high electron doping concentration, $(BHT\text{-}Ni)_h$, compared to the pristine structure. All images are at the 0.0011 $e/Å^{-3}$ isosurface value.

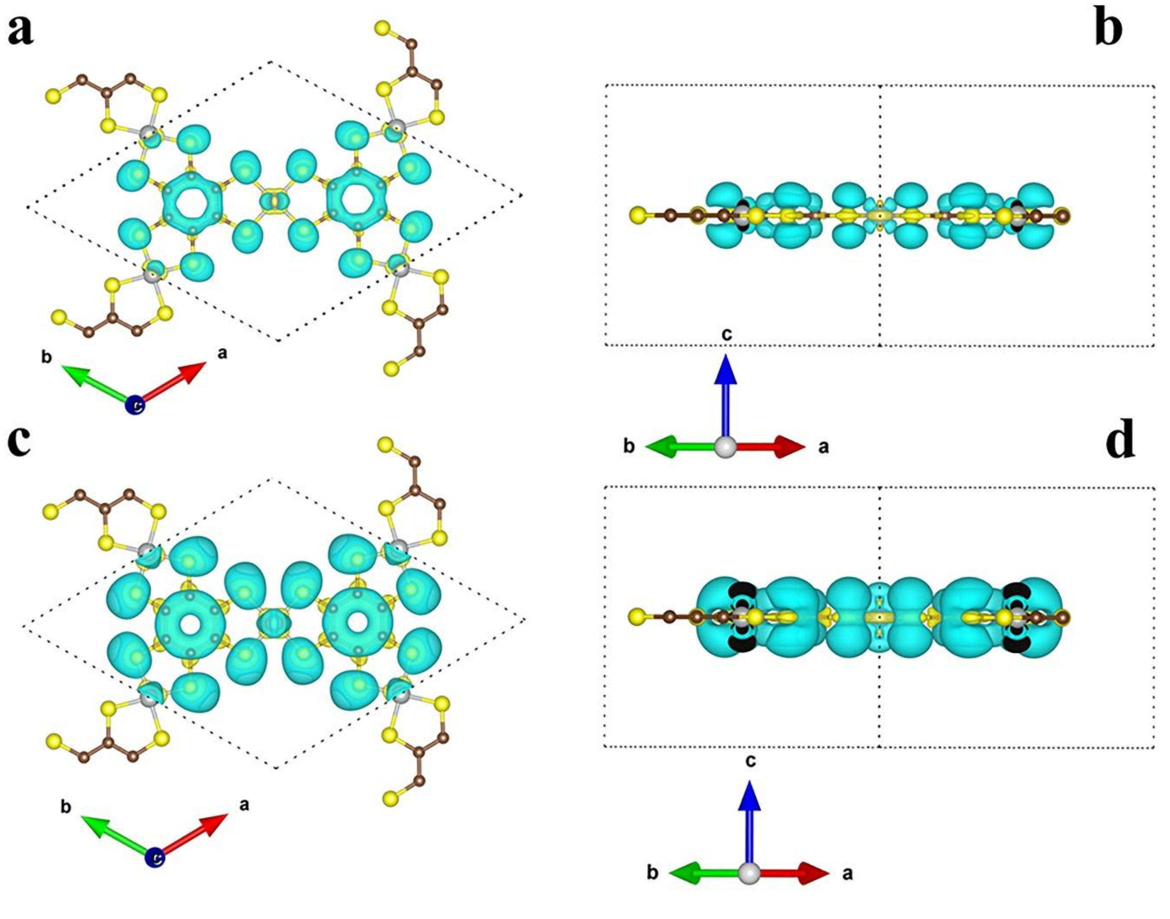

Figure S7. Spin-polarized electron density difference (δρ) of $(BHT\text{-}Ni)_h$ with an isosurface value of 0.004 $e/Å^{-3}$.

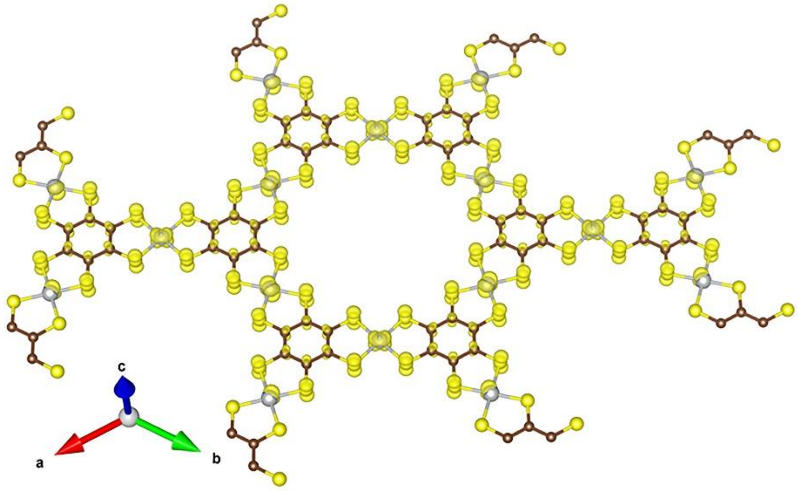

Figure S8. Zoom-in of Figure 5e shows the semi-infinite chiral edge states of the $(BHT\text{-}Ni)_h$ near the Fermi level. (a) For the left side. (b) For the right side of the $(BHT\text{-}Ni)_h$.

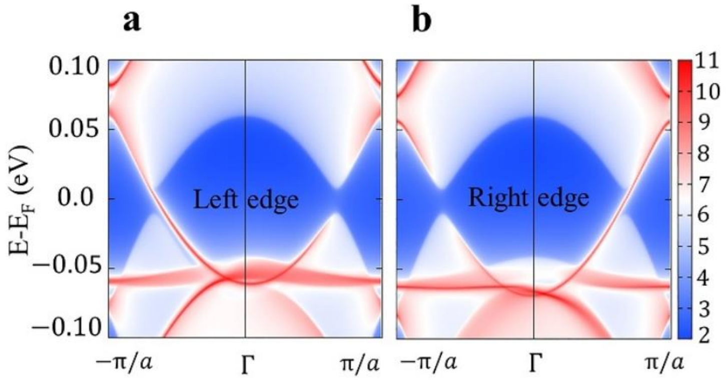

Figure S9. Projected density of states of (a) $(BHT\text{-}Ni)_p$, (b) *trans*-$(BHT\text{-}Ni)_p$ and (c) *cis*-$(BHT\text{-}Ni)_p$. The Kagome bands above the Fermi level are mostly contributed by S p_z orbitals in **a**, and Se and S p_z orbitals in **b** and **c**.

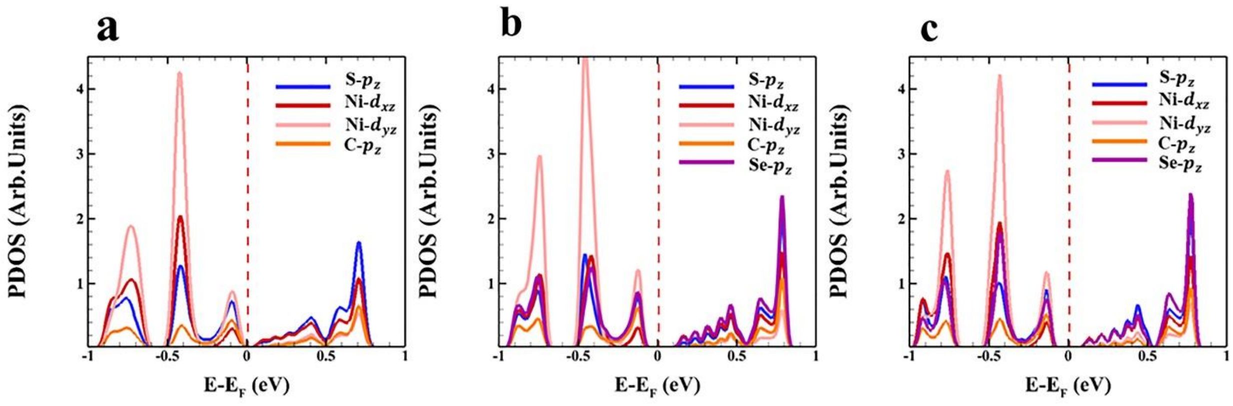

Figure S10. Band structure of *cis*- and *trans*-$(BHT\text{-}Ni)_p$. (a) Band structure of the *trans*-$(BHT\text{-}Ni)_p$ and (b) the *cis*-$(BHT\text{-}Ni)_p$ without considering SOC. Band structures of (c) *trans*- and (d) *cis*-$(BHT\text{-}Ni)_p$ with considering SOC. Δ_1 is the Dirac gap, Δ_2 and Δ_3 are local and global gaps at Γ point, respectively.

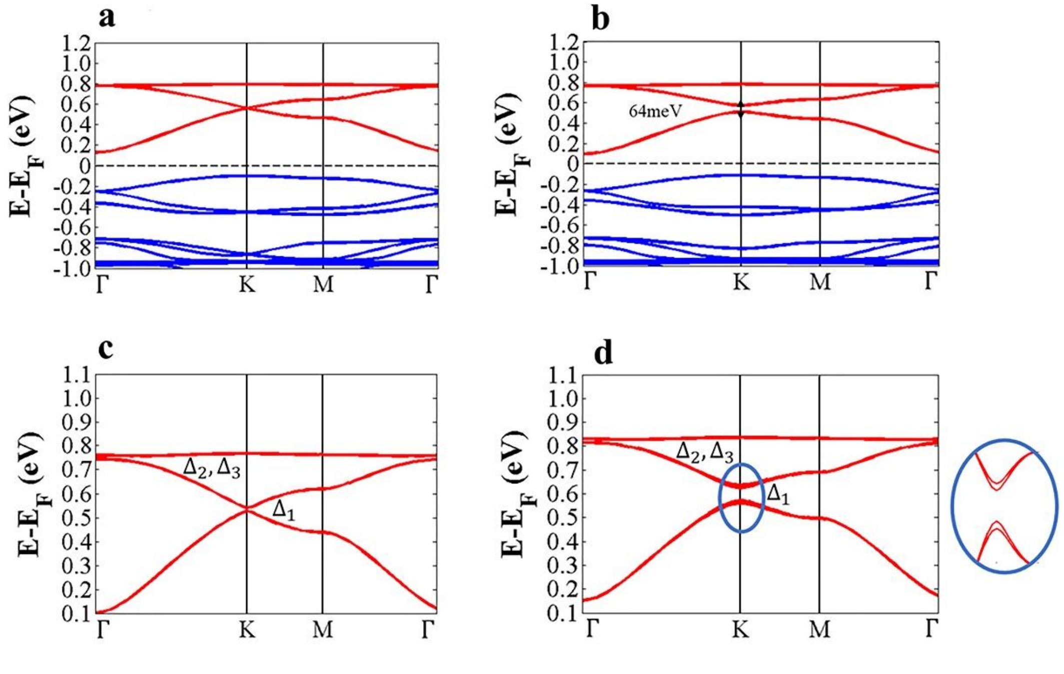

Figure S11. (a) Band structure of the *trans*-(BHT-Ni)$_l$ with considering SOC. (b) The σ_{xy}^{z} tensor element of SHC as a function of the E_F position for the *trans*-(BHT-Ni)$_l$ sheet, showing its maximum value at the Fermi level.

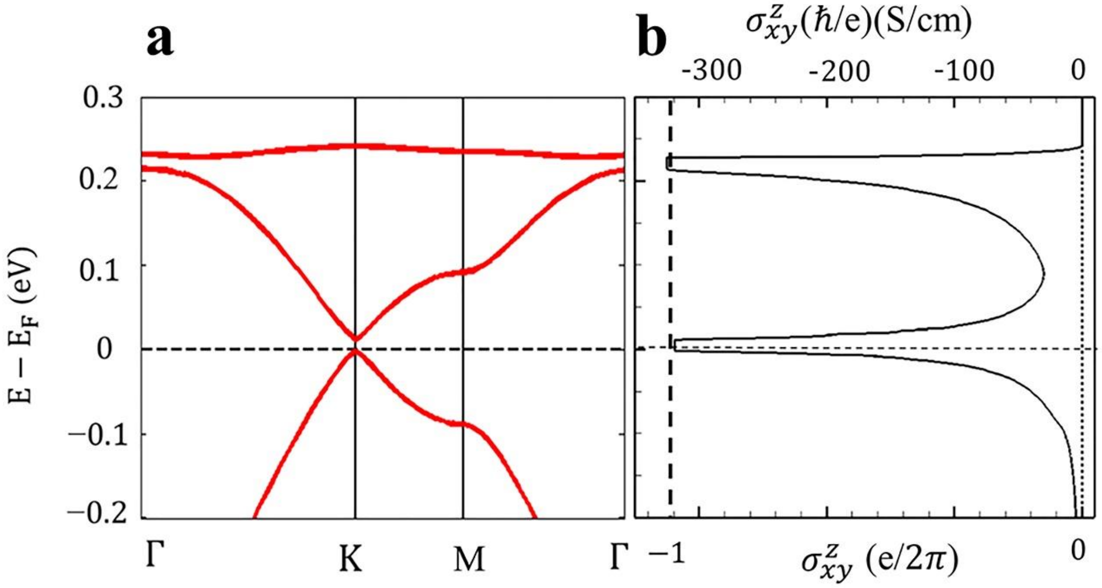

Table S3. Comparison of SOC gaps in pristine structures (denoted as p) and those with low electron doping (denoted as l).

Structure	(Δ_1)	(Δ_2)	(Δ_3)
(BHT-Ni)$_p$	14	17	5.4
cis-(BHT-Ni)$_p$	52	15.9	12.5
trans-(BHT-Ni)$_p$	12.6	16.3	12.9
(BHT-Ni)$_l$	15.4	18.6	8.5
cis-(BHT-Ni)$_l$	17	17.6	14.1
trans-(BHT-Ni)$_l$	13.8	17.6	14.3

Figure S12. (a) and (b) Semi-infinite edge states of the *trans*-(BHT-Ni)$_{l}$ for the left and right sides of the sample, respectively. (c) and (d) Semi-infinite edge states of the *cis*-(BHT-Ni)$_{l}$ for the left and right sides of the sample, respectively.

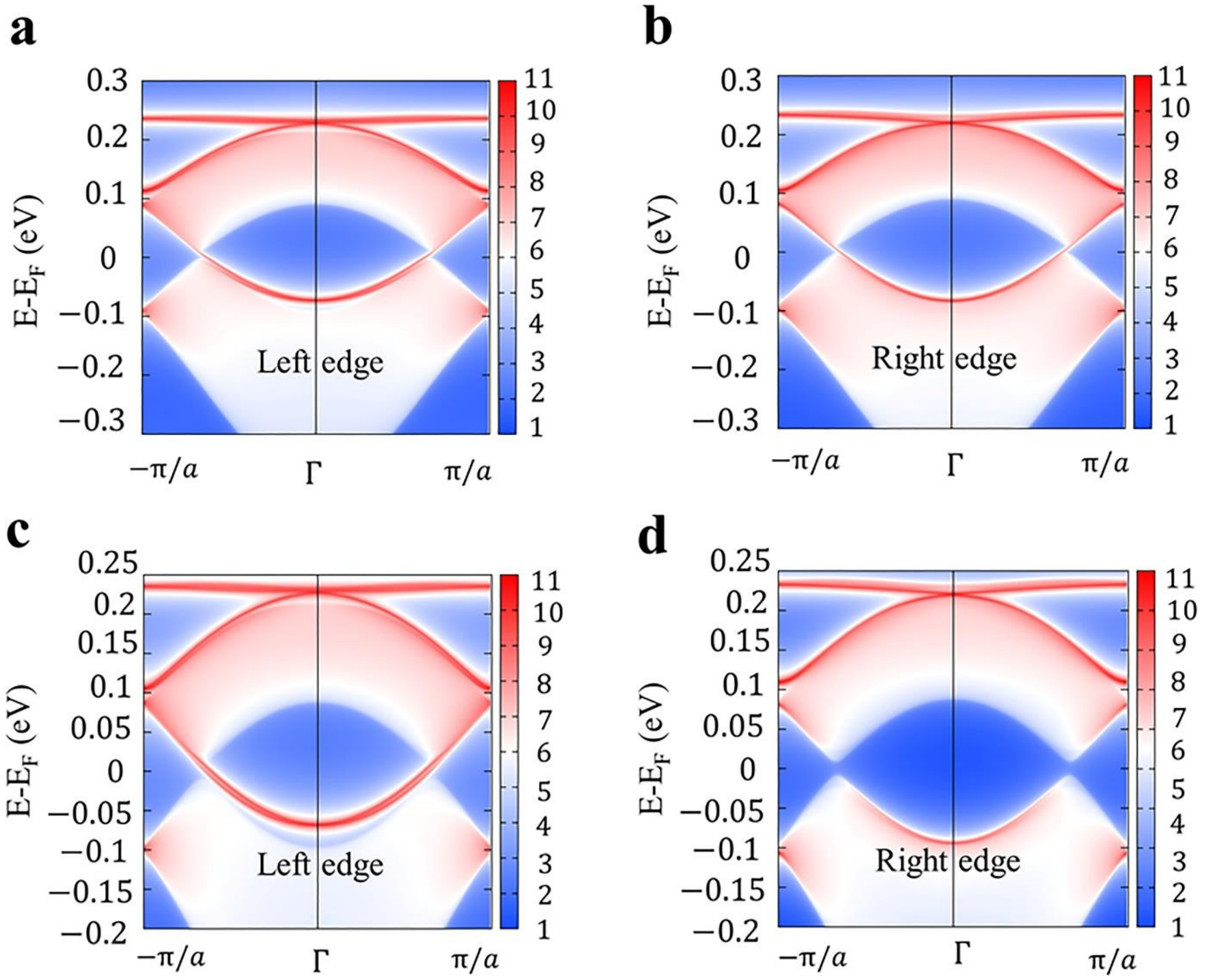

Figure S13. (a) Ordinary Berry curvature (BC) of the *cis*-(BHT-Ni)$_1$ without SOC, along the high-symmetry lines. (b) The calculated BC distribution of valence bands below the Fermi level in the ($\boldsymbol{k}_x$, $\boldsymbol{k}_y$) plane in arbitrary units.

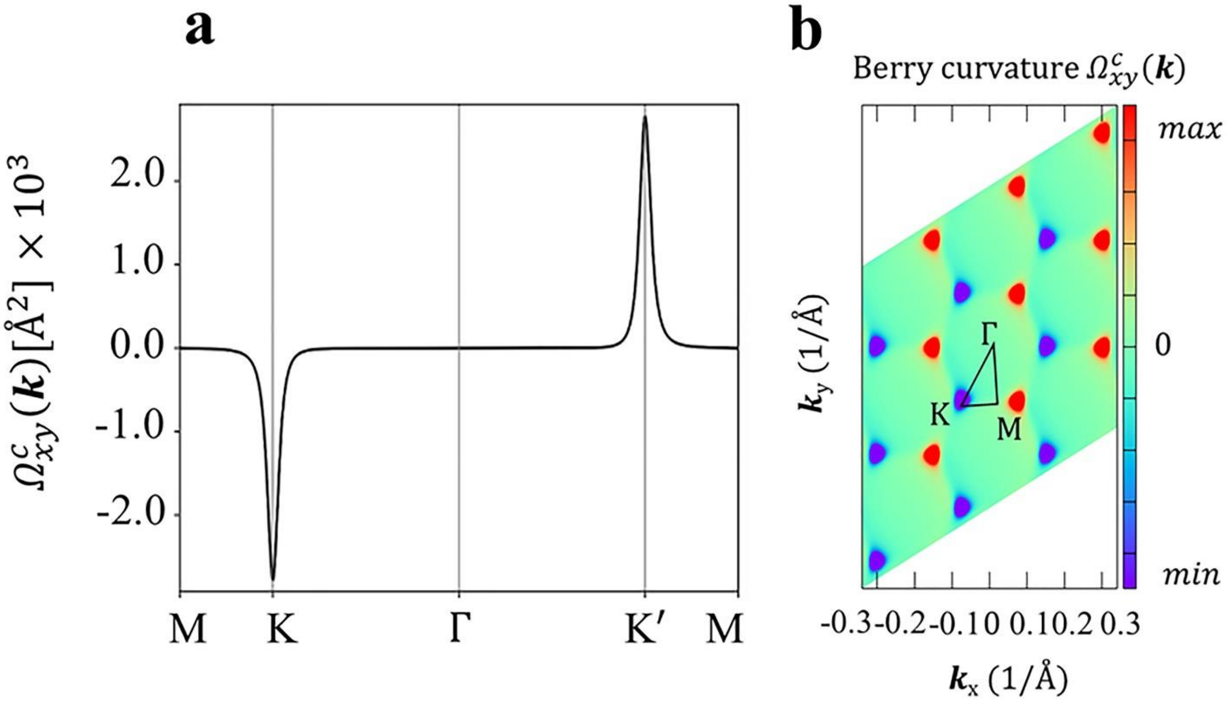

REFERENCES:

(1) Zhao, B.; Zhang, J.; Feng, W.; Yao, Y. and Yang, Z. Quantum spin Hall and Z_2 metallic states in an organic material. *Phys. Rev. B*. **2014**, 90 (20), 201403-201407. DOI: 10.1103/PhysRevB.90.201403.

(2) Liu, L.; Pai, C.F.; Li, Y.; Tseng, H.W.; Ralph, D.C. and Buhrman, R.A. Spin-torque switching with the giant spin Hall effect of tantalum. *Science*. **2012**, 336 (6081), 555-558. DOI: 10.1126/science.1218197.

(3) Pai, C.F.; Liu, L.; Li, Y.; Tseng, H.W.; Ralph, D.C. and Buhrman, R.A. Spin transfer torque devices utilizing the giant spin Hall effect of tungsten. *Appl. Phys. Lett.* **2012**, 101 (12), 122404-122407. DOI: 10.1063/1.4753947.

(4) Liu, L.; Moriyama, T.; Ralph, D.C. and Buhrman, R.A. Spin-torque ferromagnetic resonance induced by the spin Hall effect. *Phys. Rev. Lett.* **2011**, 106 (3), 036601-036604. DOI: 10.1103/PhysRevLett.106.036601.

(5) Wang, Y.; Deorani, P.; Qiu, X.; Kwon, J.H. and Yang, H. Determination of intrinsic spin Hall angle in Pt. *Appl. Phys. Lett.* **2014**, 105 (15), 152412-152415. DOI: 10.1063/1.4898593.

(6) Ng, T.; Luo, Y.; Yuan, J.; Wu, Y.; Yang, H. and Shen, L. Origin and enhancement of the spin Hall angle in the Weyl semimetals LaAlSi and LaAlGe. *Phys. Rev. B*. **2021**, 104 (1), 014412-014420. DOI: 10.1103/PhysRevB.104.014412.

(7) Qiao, J.; Zhou, J.; Yuan, Z. and Zhao, W. Calculation of intrinsic spin Hall conductivity by Wannier interpolation. *Phys. Rev. B*. **2018**, 98 (21), 214402-214411. DOI: 10.1103/PhysRevB.98.214402.

(8) Farzaneh, S.M. and Rakheja, S. Intrinsic spin Hall effect in topological insulators: A first-principles study. *Phys. Rev. Mater.* **2020**, 4 (11), 114202-114210. DOI: 10.1103/PhysRevMaterials.4.114202.

(9) Mellnik, A.R.; Lee, J.S.; Richardella, A.; Grab, J.L.; Mintun, P.J.; Fischer, M.H.; Vaezi, A.; Manchon, A.; Kim, E.A.; Samarth, N. and et al. Spin-transfer torque generated by a topological insulator. *Nature*. **2014**, 511 (7510), 449-451. DOI: 10.1038/nature13534.

(10) Khang, N.H.D.; Ueda, Y. and Hai, P.N. A conductive topological insulator with large spin Hall effect for ultralow power spin–orbit torque switching. *Nat. Mater.* **2018**, 17 (9), 808-813. DOI: 10.1038/s41563-018-0137-y.

(11) Fan, Y.; Upadhyaya, P.; Kou, X.; Lang, M.; Takei, S.; Wang, Z.; Tang, J.; He, L.; Chang, L.T.; Montazeri, M. and et al. Magnetization switching through giant spin–orbit torque in a magnetically doped topological insulator heterostructure. *Nat. Mater.* **2014**, 13 (7), 699-704. DOI: 10.1038/nmat3973.

(12) Kondou, K.; Yoshimi, R.; Tsukazaki, A.; Fukuma, Y.; Matsuno, J.; Takahashi, K.S.; Kawasaki, M.; Tokura, Y. and Otani, Y. Fermi-level-dependent charge-to-spin current conversion by Dirac surface states of topological insulators. *Nat. Phys*. **2016**, 12 (11), 1027-1031. DOI: 10.1038/nphys3833.

(13) Yasuda, K.; Tsukazaki, A.; Yoshimi, R.; Kondou, K.; Takahashi, K.S.; Otani, Y.; Kawasaki, M. and Tokura, Y. Current-nonlinear Hall effect and spin-orbit torque magnetization switching in a magnetic topological insulator. *Phys. Rev. Lett.* **2017,** 119 (13), 137204-137208. DOI: 10.1103/PhysRevLett.119.137204.

(14) Zhao, N.N.; Liu, K. and Lu, Z.Y. Large intrinsic spin Hall conductivity and spin Hall angle in the nodal-line semimetals $ThAl_2$ and $ThGa_2$. *Phys. Rev. B.* **2022**, 105 (23), 235119-235124. DOI: 10.1103/PhysRevB.105.235119.

(15) Shi, S.; Liang, S.; Zhu, Z.; Cai, K.; Pollard, S.D.; Wang, Y.; Wang, J.; Wang, Q.; He, P.; Yu, J. and et al. All-electric magnetization switching and Dzyaloshinskii–Moriya interaction in WTe_2/ferromagnet heterostructures. *Nat. Nanotechnol.* **2019**, 14 (10), 945-949. DOI: 10.1038/s41565-019-0525-8.

(16) MacNeill, D.; Stiehl, G.M.; Guimaraes, M.H.D.; Buhrman, R.A.; Park, J. and Ralph, D.C. Control of spin–orbit torques through crystal symmetry in WTe_2/ferromagnet bilayers. *Nat. Phys.* **2017**, 13 (3), 300-305. DOI: 10.1038/nphys3933.

(17) Zhao, B.; Khokhriakov, D.; Zhang, Y.; Fu, H.; Karpiak, B.; Hoque, A.M.; Xu, X.; Jiang, Y.; Yan, B. and Dash, S.P. Observation of charge to spin conversion in Weyl semimetal WTe_2 at room temperature. *Phys. Rev. Res.* **2020,** 2 (1), 013286-013293. DOI: 10.1103/PhysRevResearch.2.013286.

(18) Zhou, J.; Qiao, J.; Bournel, A. and Zhao, W. Intrinsic spin Hall conductivity of the semimetals $MoTe_2$ and WTe_2. *Phys. Rev. B.* **2019***,* 99 (6), 060408-060412. DOI: 10.1103/PhysRevB.99.060408.

(19) Stiehl, G.M.; Li, R.; Gupta, V.; Baggari, I.E.; Jiang, S.; Xie, H.; Kourkoutis, L.F.; Mak, K.F.; Shan, J.; Buhrman, R.A. and et al. Layer-dependent spin-orbit torques generated by the

centrosymmetric transition metal dichalcogenide β-$MoTe_2$. *Phys. Rev. B.* **2019**, 100 (18), 184402-184413. DOI: 10.1103/PhysRevB.100.184402.
(20) Sun, Y.; Zhang, Y.; Felser, C. and Yan, B. Strong intrinsic spin Hall effect in the TaAs family of Weyl semimetals. *Phys. Rev. Lett.* **2016**, 117 (14), 146403-146407. DOI: 10.1103/PhysRevLett.117.146403.
